# Shaping the “hot” immunogenic tumor microenvironment by nanoparticles co‐delivering oncolytic peptide and TGF‐β1 siRNA for boosting checkpoint blockade therapy

**DOI:** 10.1002/btm2.10392

**Published:** 2022-08-11

**Authors:** Cao Dai Phung, Bao Loc Nguyen, Jee‐Heon Jeong, Jae‐Hoon Chang, Sung Giu Jin, Han‐Gon Choi, Sae Kwang Ku, Jong Oh Kim

**Affiliations:** ^1^ College of Pharmacy Yeungnam University Gyeongsan Republic of Korea; ^2^ Department of Precision Medicine, School of Medicine Sungkyunkwan University Suwon Republic of Korea; ^3^ Department of Pharmaceutical Engineering Dankook University Cheonan Republic of Korea; ^4^ College of Pharmacy & Institute of Pharmaceutical Science and Technology Hanyang University Ansan Republic of Korea; ^5^ College of Korean Medicine Daegu Haany University Gyeongsan Republic of Korea

**Keywords:** cancer immunotherapy, hybrid nanoparticle, LTX‐315, tumor microenvironment

## Abstract

Induction of potent immune responses toward tumors remains challenging in cancer immunotherapy, in which it only showed benefits in a minority of patients with “hot” tumors, which possess pre‐existing effector immune cells within the tumor. In this study, we proposed a nanoparticle‐based strategy to fire up the “cold” tumor by upregulating the components associated with T and NK cell recruitment and activation and suppressing TGF‐β1 secretion by tumor cells. Specifically, LTX‐315, a first‐in‐class oncolytic cationic peptide, and TGF‐β1 siRNA were co‐entrapped in a polymer‐lipid hybrid nanoparticle comprising PLGA, DSPE‐mPEG, and DSPE‐PEG‐conjugated with cRGD peptide (LTX/siR‐NPs). The LTX/siR‐NPs showed significant inhibition of TGF‐β1 expression, induction of type I interferon release, and triggering immunogenic cell death (ICD) in treated tumor cells, indicated via the increased levels of danger molecules, an in vitro setting. The in vivo data showed that the LTX/siR‐NPs could effectively protect the LTX‐315 peptide from degradation in serum, which highly accumulated in tumor tissue. Consequently, the LTX/siR‐NPs robustly suppressed TGF‐β1 production by tumor cells and created an immunologically active tumor with high infiltration of antitumor effector immune cells. As a result, the combination of LTX/siR‐NP treatment with NKG2A checkpoint inhibitor therapy remarkably increased numbers of CD8^+^NKG2D^+^ and NK1.1^+^NKG2D^+^ within tumor masses, and importantly, inhibited the tumor growth and prolonged survival rate of treated mice. Taken together, this study suggests the potential of the LTX/siR‐NPs for inflaming the “cold” tumor for potentiating the efficacy of cancer immunotherapy.

## 1 INTRODUCTION

The reduced expression of tumor antigens and the upregulation of anti‐apoptotic molecules by the tumor cells, together with the formation of the immunosuppressive tumor microenvironment (TME) consisting of numerous immunosuppressive factors, such as transforming growth factor‐beta (TGF‐β), IL‐10, regulatory T cells, and immune checkpoint components, are considered as major reasons for the exhaustion of effector lymphocytes, resulting in the immune evasion of the tumors.^1^, ^2^, ^3^ Recently, blocking immune checkpoint molecules, such as PD‐1/PD‐L1 and CTLA‐4, have shown significant efficacy against several types of tumors via restoring T cell antitumor function.^4^, ^5^, ^6^ However, the immune checkpoint inhibitors have shown to be ineffective in “cold” tumors, which lack pre‐existing intratumoral lymphocytes, such as breast, ovarian, and pancreatic carcinoma.^7^ Moreover, a substantial fraction of cancer patients with an initial response was found to develop acquired resistance to this therapeutic strategy.^8^ Recent studies revealed that a majority of tumor‐infiltrated NK cells and CD8^+^ T cells express the NKG2A receptor that transmits the inhibitory signal to inhibit the function of NK and CD8^+^ T cells upon binding with HLA‐E in human or Qa‐1^b^ ligand in mice.^9^, ^10^ Having established that numerous tumor types upregulate HLA‐E/Qa‐1^b^ levels as a mechanism to resist the killing of NK and T cells, NKG2A checkpoint inhibitors have been developed and demonstrated to unleash their cytotoxic activity against tumors in both preclinical and clinical investigations.^11^

Recently, several cationic antimicrobial peptides (CAPs), such as LTX‐315, have been extensively investigated as novel anticancer agents due to their ability to kill tumor cells and create a highly proinflammatory status of the TME, mainly via causing the destabilization of tumor cell plasma and mitochondrial membrane.^12^ Subsequently, the CAPs trigger the immunogenic cell death (ICD) in tumor cells via Bax/Bak‐1 and caspase apoptotic pathways, which is along with the exposure or release of tumor antigens, danger‐associated molecular pattern (DAMP) molecules such as calreticulin, ATP and high mobility group protein B1 (HMGB1) molecules, and type I interferons (IFNs).^13^, ^14^, ^15^ The DAMP molecules have a critical role in activating the antigen‐presenting cells (APCs) such as dendritic cells (DCs) in the TME through stimulating their specific and this finally activated the antitumor adaptive immunity.^15^ Moreover, evidence from both preclinical and clinical investigations indicates that local injection of LTX‐315 could robustly reshape the “cold” TME into a “hot” state by enhanced recruitment and stimulation of phagocytic cells, neutrophils, lymphocytes, and other effector immune cells,^16^, ^17^ while decreasing immunosuppressive cells.^18^ However, this peptide is rapidly degraded into nontoxic metabolites and eliminated upon systemic administration due to its high serum protein binding ability.^19^ Therefore, the application of LTX‐315 is limited to only accessible tumors, such as melanoma, via intratumoral injection.

Since transforming growth factor‐beta 1 (TGF‐β1) has been identified as one of the critical factors of bad prognosis in many tumor types,^20^ TGF‐β1 blockades have emerged as a potential cancer therapy strategy. The overproduction of TGF‐β1 in the formation of a favorable microenvironment for tumor cell proliferation and metastasis, including mitigating the function of effector immune cells, promoting the generation of immunosuppressive cells, such as regulatory T cells (Tregs) and M_2_‐like tumor‐associated macrophages, facilitating the angiogenesis, and increasing the production of collagen, the main component of extracellular matrix in the tumors.^21^, ^22^ As such, TGF‐β1 suppression could inhibit the tumor cell proliferation, restore the antitumor activity of cytotoxic lymphocytes, and reduce the collagen matrix, leading to the enhanced infiltration of CD8^+^ T cells and penetration of both chemotherapeutic drugs and nanoparticles into solid tumors.^23^, ^24^, ^25^ Integrins, the extracellular matrix receptors consisting of α and β subunits, are well‐documented for their essential in all tumor progression stages and are identified as a hallmark of cancers. Among identified integrin receptors, αvβ3 has been demonstrated to be the most critical integrin for angiogenesis and upregulated in activated endothelial cells as well as several tumor cells, such as melanoma, glioblastoma, pancreatic, colon, and breast carcinoma, while absent in resting endothelial cells and normal tissues, suggesting it as a suitable marker for tumor‐targeted therapy.^26^ Cyclic Arg‐Gly‐Asp (cRGD) peptide is reported to preferentially bind to αvβ3 integrin and exert a remarkably antiangiogenic effect as a result of the blockade of the interaction between αvβ3 and its corresponding extracellular matrix ligands.^27^, ^28^

**SCHEME 1:**
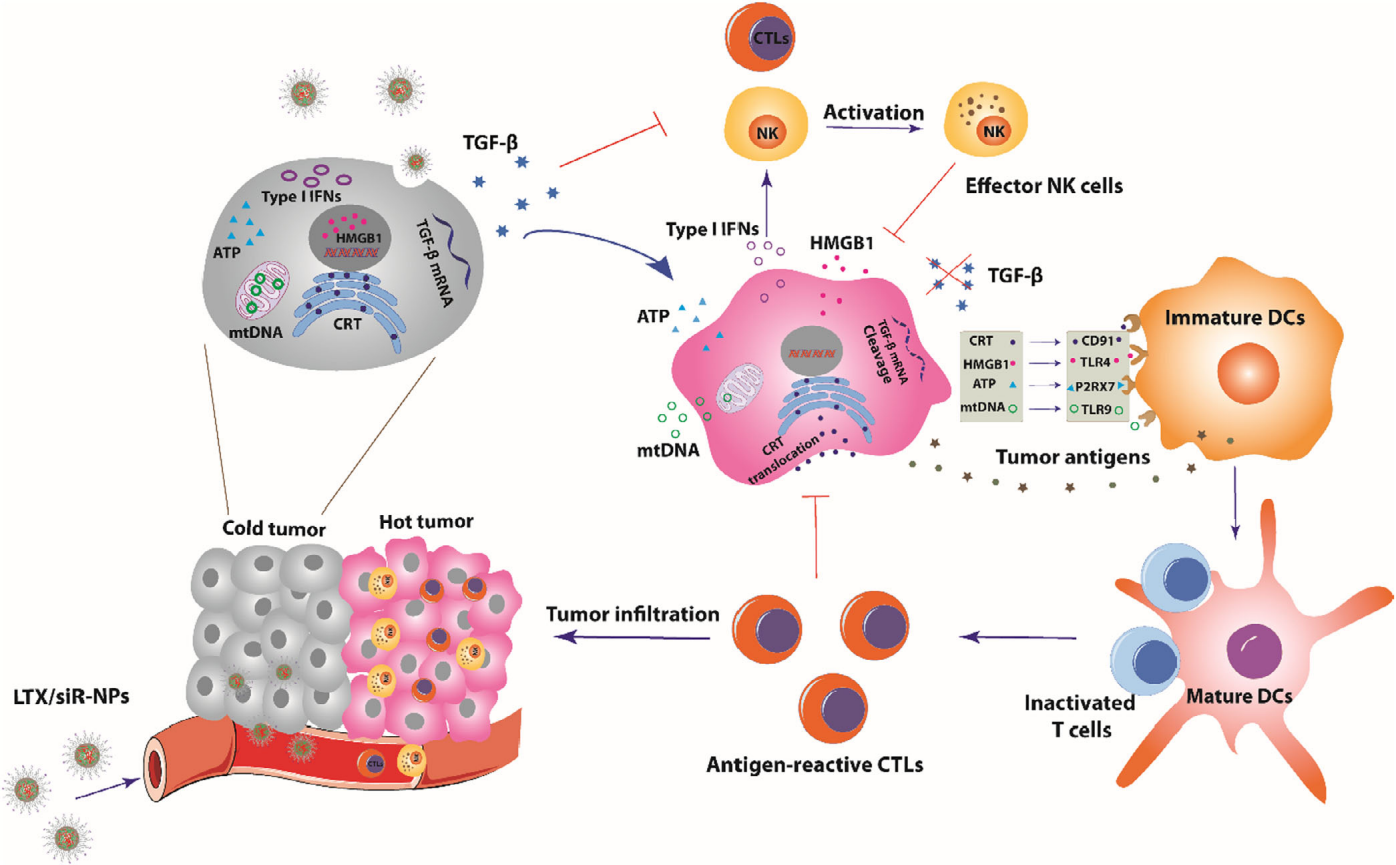
Schematic illustration of the mechanism of action of LTX/siR‐NPs in shaping the “hot” immunogenic tumor microenvironment

**FIGURE 1:**
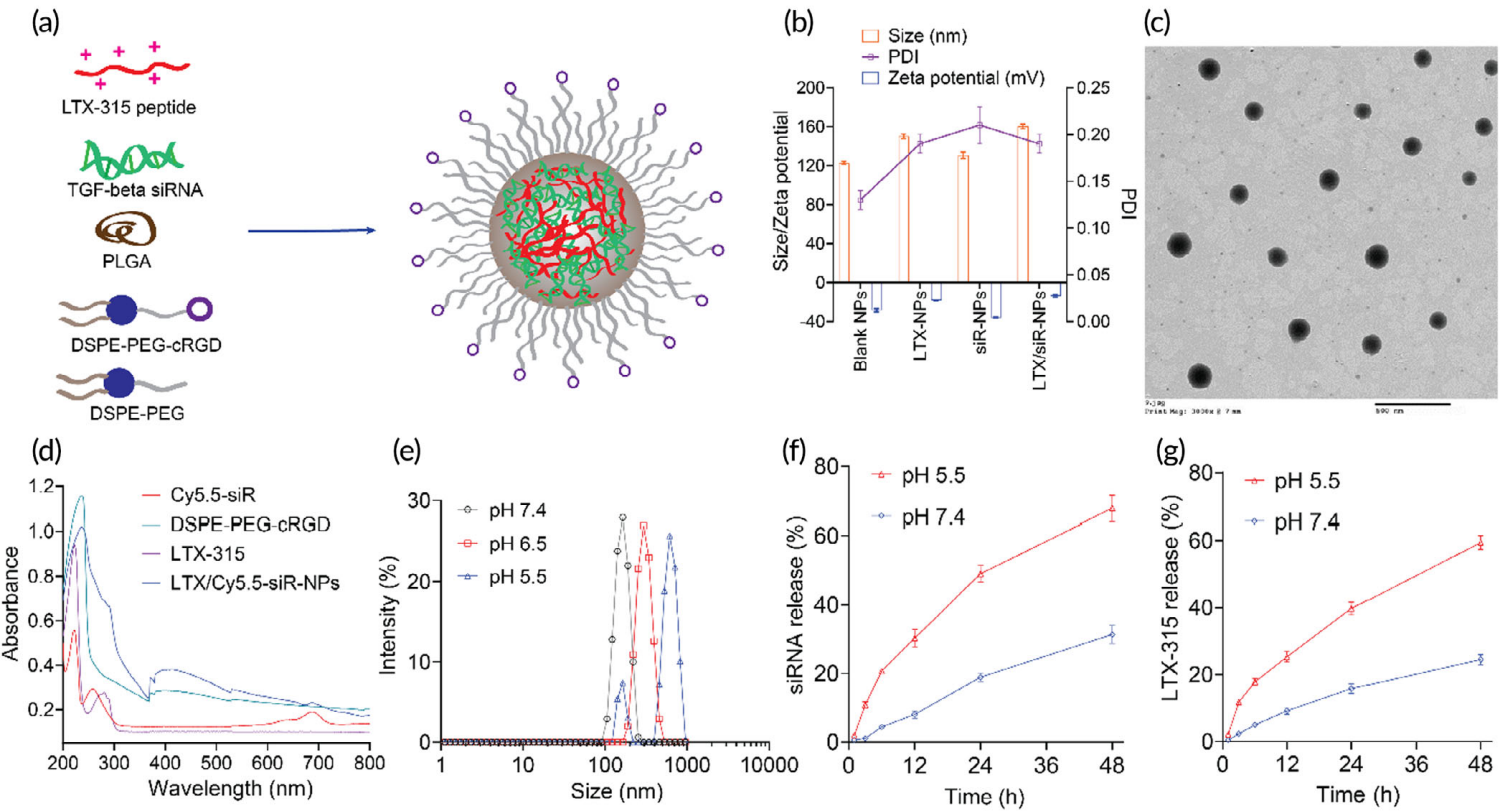
LTX/siR‐NPs preparation and characterization. (a) The representative structure of LTX/siR‐NPs. (b) Particle size distribution and zeta potential of LTX/siR‐NPs were measured by the dynamic light scattering method. (c) The spherical morphology of LTX/siR‐NPs was observed by transmission electron microscopy (scale bar: 500 nm). (d) The UV–VIS spectra of components and LTX/siR‐NPs confirmed the successful load of LTX‐315 and TGF‐β siRNA into the hybrid nanoparticles. (e) The influence of pH conditions on the size of LTX/siR‐NPs. The in vitro accumulative drug‐release profile of (f) siRNA and (g) LTX‐315 at different pH conditions

In this work, we address the hurdles hampering the immune responses toward the poorly immunogenic tumor by combining a cRGD‐linked polymer‐lipid hybrid nanoparticle co‐delivering LTX‐315 peptide and TGF‐β1 siRNA (LTX/siR‐NPs) with an NKG2A inhibitor (Scheme 1, Figure 1a). We hypothesized that the LTX/siR‐NPs could enhance the antitumor activity of LTX‐315 and the siRNA upon systemic administration, and consequently upregulate the components associated with T and NK cell recruitment and activation as well as effectively suppress TGF‐β1 secretion by tumor cells, shaping an immunologically “hot” tumor with high infiltration of lymphocytes. Simultaneously, the combination regimen of LTX/siR‐NPs with anti‐NKG2A antibody exhibits a superior potency in enhancing NK and CD8^+^ T cells' cytotoxic function against the tumor cells, and as a result, inhibits the tumor growth and prolongs the survival rate of treated mice. Taken together, we investigated the potential of the LTX/siR‐NPs to inflame the “cold” tumor for potentiating the efficacy of cancer immunotherapy.

## 2 RESULTS AND DISCUSSION

### 2.1 Preparation of LTX/siR‐NPs for systemic delivery of LTX‐315 and TGF‐β1 siRNA

We designed a polymer‐lipid hybrid nanoplatform (Figure 1a) for co‐delivery of LTX‐315 peptide and TGF‐β1 siRNA to the tumor. Owing to the highly cationic charge in nature, LTX‐315 peptide could electrostatically complex with TGF‐β1 siRNA and facilitate the cytosolic siRNA delivery.^4^, ^29^ The LTX‐315/siRNA complex was then entrapped into the PLGA core, followed by coating with a mixture of lipids containing 1,2‐distearoyl‐sn‐glycero‐3‐phosphoethanolamine‐N‐[methoxy(polyethylene glycol)‐2000] (DSPE‐mPEG) and Cyclo (Arg‐Gly‐Asp‐D‐Phe‐Cys)‐conjugated 1,2‐distearoyl‐sn‐glycero‐3‐phosphoethanolamine‐N‐[maleimide(polyethylene glycol)‐2000] (DSPE‐PEG‐cRGD) to prolong the circulation time and improve cancer cell uptake. As shown in Figure S2a, the siRNA was completely condensed with LTX‐315 peptide at an N/P ratio of 10. We formulated the hybrid NPs at a lipids/PLGA ratio of 0.5 to achieve a uniformed and small size of NPs, as followed in the previous report.^30^ As characterized by the dynamic light scattering (DLS) method (Figure 1b), the blank NPs were formed at an average size of ~120 nm, and the addition of LTX‐315 and siRNA showed an increased particle size of LTX/siR‐NPs (~160 nm). The transmission electron microscopy (TEM) characterization showed a spherical morphology and core–shell structure of the LTX/siR‐NPs (Figure 1c). We further examined the successful formation of the LTX/siR‐NPs by recording the UV–VIS spectra of the LTX‐315, Cyanine 5.5‐labeled siRNA (Cy5.5‐siR), DSPE‐PEG‐cRGD, and the LTX/siR‐NPs. As shown in Figure 1d, the corresponding absorption peak of tryptophan at 280 nm in LTX‐315 and Cy5.5 at 694 nm was observed in the LTX/siR‐NP spectra, indicating the successful entrapment of the LTX‐315 peptide and siRNA into the hybrid NPs. The capability of the polymer‐lipid hybrid NPs in co‐delivery of LTX‐315 and TGF‐β1 siRNA was further confirmed by calculating the encapsulation efficiency (EE) of the cargos. Figure S2b showed the high efficiency in loading both the LTX‐315 and siRNA in the hybrid NPs owing to the highly electrostatic interaction between PLGA‐COOH, cationic LTX‐315, and siRNA.

The LTX/siR‐NPs were highly stable in the physiological condition with no significant changes in size and polydispersity index (PDI) after incubation with PBS buffer pH 7.4 supplemented with 10% serum (Figure S2c). Moreover, we measured the serum protein adsorption onto the surface of the LTX/siR‐NPs, which has been demonstrated to affect the therapeutic activity of the NPs negatively. Figure S2d showed the low amounts of adsorbed serum proteins onto the NP surface up to 24 h of incubation. We then examined the changes in the size of the LTX/siR‐NPs in different pH conditions. It is found that the size of the NPs was dramatically increased along with the decrease of pH values (Figure 1e), showing the responsiveness of the NPs to the acidic environment, probably owing to the increased penetration of proton into the NPs, resulting in the protonation of LTX‐315, consequently causing the NP swelling and destabilization. As a result, the release of LTX‐315 and siRNA was accelerated at the endolysosomal pH 5.5 compared to the physiological pH 7.4 (Figure 1f,g).

### 2.2 LTX/siR‐NPs efficiently internalized tumor cells and mediated TGF‐β1 suppression and immunogenic cell death

**FIGURE 2:**
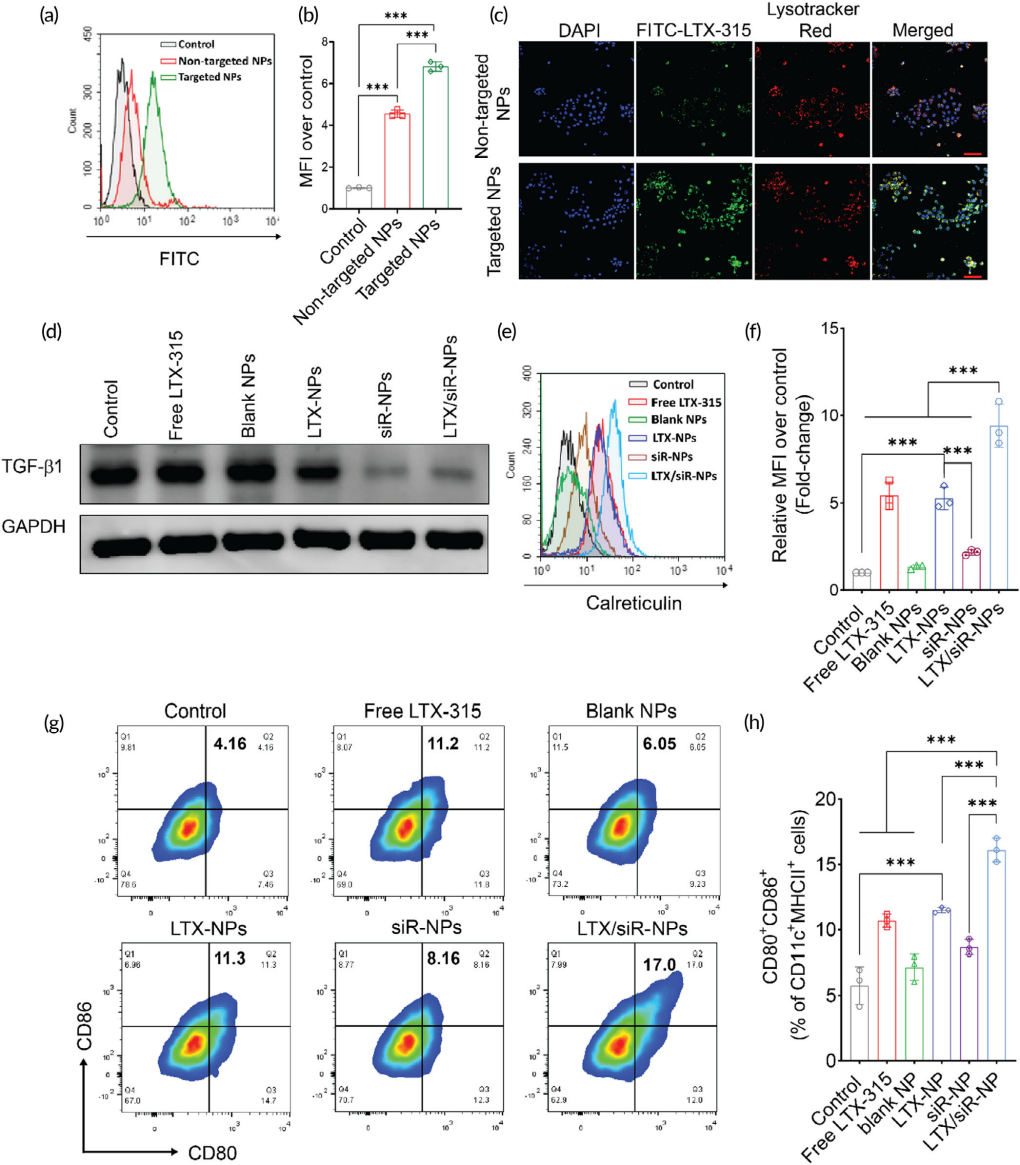
Cellular uptake of LTX/siR‐NPs into 4T1 cells followed by the induction of the immunogenic cell death and reduced expression of TGF‐β1. The internalization of targeted LTX/siR‐NPs (with cRGD conjugation) and nontargeted LTX/siR‐NPs (without cRGD conjugation) into 4T1 cells was confirmed by (a, b) flow cytometry analysis, and (c) confocal laser scanning microscopy (scale bar: 50 nm). (d) Western Blot analysis of TGF‐β1 expression in 4T1 cells after treatment with (1) PBS (Control), (2) free LTX‐315, (3) blank NPs, (4) LTX‐NPs, (5) siR‐NPs, and (6) LTX/siR‐NPs. (e, f) The plasma membrane expression of calreticulin in 4T1 cells after treatment determined by flow cytometry. (g, h) The expression of co‐stimulatory molecules on the surface of BMDCs after the treatments with supernatant of treated cancer cells. Data were presented as mean ± SD (*n* = 3). **p < 0.01; ***p < 0.001

To examine whether the cRGD modification on the surface of the LTX/siR‐NPs could promote the internalization of the NPs into the tumor cells or not, we incubated the fluorescein isothiocyanate (FITC)‐labeled LTX‐315 encapsulated NPs functionalized or nonfunctionalized with cRGD (targeted NPs and nontargeted NPs, respectively) with the αvβ3‐overexpressed 4T1 tumor cells,^31^ followed by the assessment of the intracellular uptake profiles by flow cytometry and confocal laser scanning microscopy (CLSM). Figure 2a–c clearly showed that the functionalization with cRGD peptide resulted in a remarkable increase in the internalization of the LTX/siR‐NPs into the 4T1 cells. Moreover, Figure 2c showed that at 1 h post‐incubation, the green fluorescence of FITC‐LTX‐315 in the LTX/siR‐NPs was overlapped with the red fluorescence of Lysotracker Red, which selectively stains the endolysosomal organelles, indicating that the NPs were internalized into the cancer cells by endocytosis and localized in the endolysosomes. To observe the endolysosome escape of the siRNA, we encapsulated the fluorescein amidite (FAM)‐labeled siRNA into the hybrid NPs and incubated with the 4T1 cells for 8 h. Figure S3 clearly showed the dissociation of the siRNA from the endolysosome in the tumor cells, demonstrating the high efficiency of the LTX/siR‐NPs for gene delivery.

As the LTX/siR‐NPs displayed a high ability to deliver LTX‐315 peptide and TGF‐β1 siRNA to cancer cells, we tested the TGF‐β1 suppression as well as the induction of ICD in the 4T1 cells as a result of the NP treatment. As expected, the western blot data showed the significant inhibition of TGF‐β1 in the cells treated with NP formulations delivering the siRNA, including siR‐NPs and LTX/siR‐NPs (Figure 2d). We then evaluated the cytotoxic effect of LTX‐315 and different NP formulations on tumor cells by the MTS assay. We found that the blank NPs showed an inhibitory effect against 4T1 cells from a dose of 10 μg/ml, probably owing to the blocking of αvβ3 integrin receptor on the 4T1 surface by the cRGD‐functionalized on the surface of the NPs (Figure S4a). We also observed that the treatment with siR‐NPs at a siRNA concentration of 25 nM led to a significant decrease in tumor cell viability (Figure S4b), which is in agreement with previous findings that TGF‐β1 inhibition induces tumor cell death via the SAPK signaling pathway.^32^ Moreover, a markedly enhanced antitumor effect was exhibited in the hybrid NPs co‐delivery of LTX‐315 and TGF‐β1 siRNA from an equivalent LTX‐315 dose of 10 μg/ml, as compared to both free LTX‐315 and LTX‐NPs (Figure S4c–e).

Since the free LTX/siR‐NPs exerted a superior antitumor effect, we examined the induction of the ICD effect in 4T1 cells via measuring levels of DAMP molecules, including the translocation of calreticulin (CRT), and the release of ATP and high mobility group protein B1 (HMGB1) in treated tumor cells. It is well‐established that within 1 h of exposure to cytotoxic agents, CRT, the “eat‐me” signal for phagocytic cells, is upregulated and translocated to the tumor cell membrane. By 18 h, ATP, and HMGB1, the “find‐me” signal for DCs, are robustly released by the dying tumor cells, subsequently promoting the recruitment and maturation of DCs at tumor sites. As the hybrid NPs co‐loading LTX‐315 and siRNA showed enhanced antitumor activity, the highest CRT translocation (Figure 2e,f) and ATP secretion (Figure S4f) were detected in the LTX/siR‐NP‐treated cells, as measured by flow cytometric and bioluminescence assay, respectively. Furthermore, we analyzed the expressions of cytoplasmic ATP and nuclear HMGB1 by staining the treated tumor cells with Quinacrine, a fluorescence agent that selectively binds to ATP and phycoerythrin (PE)‐conjugated HMGB1 antibody, followed by observation by CLSM. Figure S4g clearly showed that the LTX/siR‐NPs provoked an increased depletion of intracellular Quinacrine and intranuclear HMGB1, indicating the stimulated release of these DC‐stimuli molecules by tumor cells to the surrounding milieu.

We then examined whether the DAMPs released from dying tumor cells could initiate the antitumor immune responses. The 4T1 cells were first treated with free LTX‐315 and several hybrid NP formulations. The culture media from treated 4T1 cells were collected and centrifuged to remove the remaining NPs completely, followed by adding to BMDCs. The maturation state of BMDCs was next characterized via analyzing the expression of CD80 and CD86, the markers for DC activation, by flow cytometry. Consistent with the finding that the NPs co‐delivering LTX‐315 and TGF‐β1 siRNA strongly induced the ICD effect in tumor cells followed by the robust release of DAMPs to culture media, the BMDCs incubated with supernatant from LTX/siR‐NPs‐treated 4T1 cells yielded the highest ratio of CD80, CD86 double‐positive cells (Figure 2g,h). Moreover, we assessed the expressions of pro‐inflammatory cytokines RNA in treated BMDCs. As shown in Figure S5b, treatment of 4T1 cells with LTX‐NPs improved the *Il‐6* gene expression in BMDCs compared to control and those treated with free LTX‐315, while the combination of LTX‐315 and TGF‐β1 siRNA provoked the expression levels of both *Il‐6* and *Tnf‐α*. These results suggest the potential of LTX/siR‐NPs in the initiation of the cancer immunity cycle, which starts with the induction of ICD in tumor cells.

### 2.3 LTX/siR‐NPs efficiently accumulated at the tumor site and fired up the poorly immunogenic tumor

**FIGURE 3:**
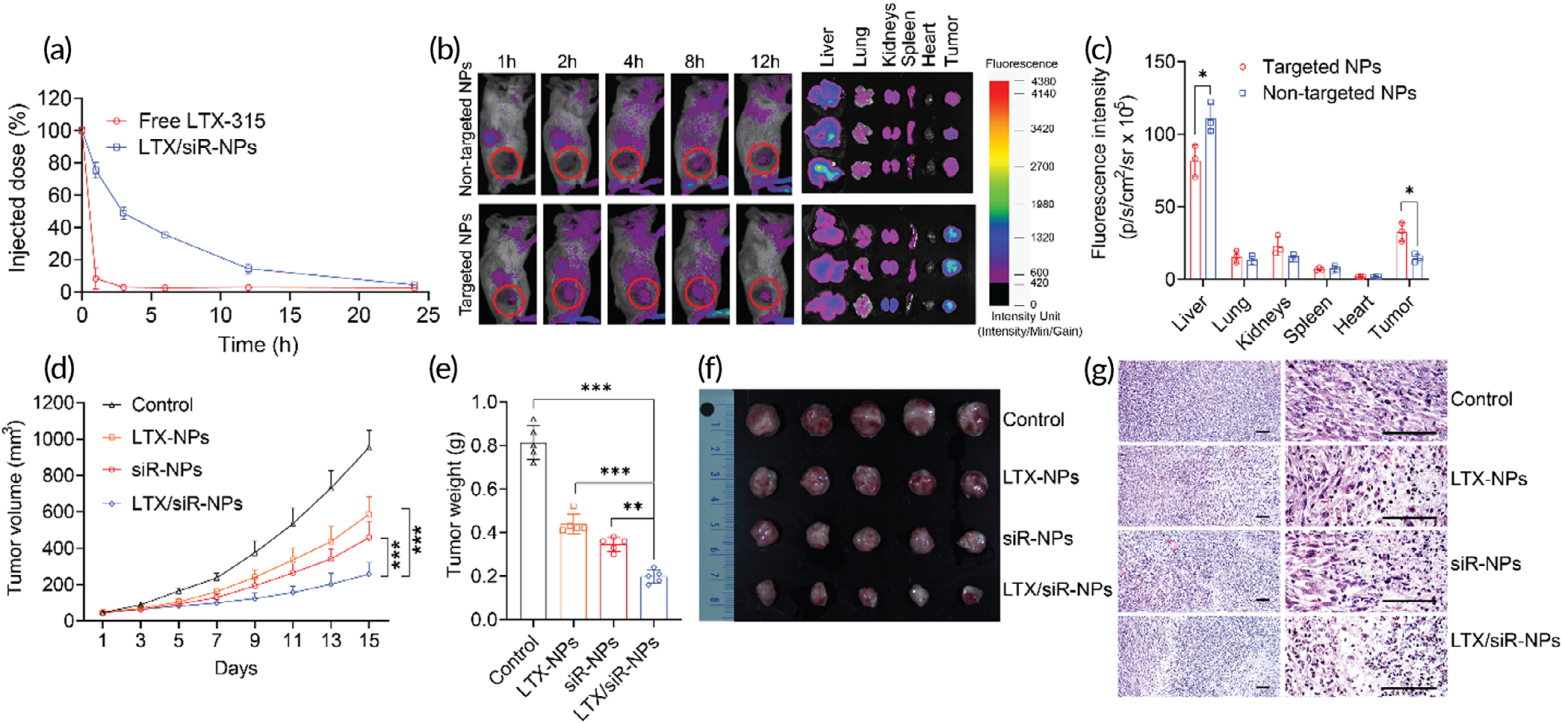
Hybrid nanoparticles prolong the circulation time, improve the accumulation at the tumor, and antitumor efficacy of loading cargoes. (a) The plasma pharmacokinetic profiles of LTX‐315 by intravenous administration of free LTX‐315 and LTX‐315 loaded NPs. (b) Fluorescent images of mice and major organs were collected from mice injected with hybrid NPs conjugated with cRGD (targeted NPs) or nontargeted NPs co‐entrapping LTX‐315 and Cyanine 5.5‐labeled siRNA. (c) Quantification of fluorescence intensities of targeted NPs and nontargeted NPs in tumors and organs of treated mice. Data were represented as mean ± SD (*n* = 3). Two‐tailed Student's *t‐*test, *p < 0.05. (d) Tumor volumes of mice treated with PBS (control), LTX‐NPs, siR‐NPs, and LTX/siR‐NPs with increasing time. Data were represented as mean ± SD (*n* = 5), two‐way ANOVA with Tukey's multiple comparison test of tumor sizes at Day 15, ***p < 0.001. (e) Tumor weight, (f) images of isolated tumors, and (g) histological analysis of tumors collected from treated mice. Data represented as mean ± SD (*n* = 5). One‐way ANOVA with Tukey's multiple comparison test, **p < 0.01; ***p < 0.001

Given the fact that the LTX‐315 is quickly eliminated upon systemic administration due to its high binding with serum proteins, we thus evaluated the capability of the hybrid NPs with a lipid‐PEG layer in prolonging the circulation time of LTX‐315 by intravenously (i.v) injecting the FITC‐labeled LTX‐315 (FITC‐LTX) and FITC‐LTX/siR‐NPs into the Balb/c mice. Figure 3a clearly showed that the free FITC‐LTX was eliminated within few minutes, while the hybrid NPs could effectively extend the half‐life of LTX‐315 peptide (*t*_1/2_ ≈ 3 h), demonstrating that the polymer‐lipid NP is a suitable delivery platform for systemic administration of LTX‐315 peptide. We next examined whether the cRGD modification could enhance the tumor accumulation of NPs. Cyanine 5.5 (Cy5.5)‐conjugated siRNA was used to form the targeted NPs (with cRGD functionalization) and nontargeted NPs (without cRGD functionalization) for tracking the biodistribution of the NPs upon systemic delivery into the 4T1 tumor‐bearing Balb/c mice. As shown in Figure 3b, the targeted NPs accumulated in the tumor tissue at a significantly higher degree than the nontargeted NPs at all time points. Ex vivo images (Figure 3b) and fluorescence quantification (Figure 3c) of tumors and major organs collected at 24 h post‐administration of NPs further confirmed that modification of the hybrid NPs with cRGD peptide resulted in a much higher NP transport into the tumors (*p* < 0.05) and significantly reduced liver accumulation of the NPs (*p* < 0.05). As the tumor homing cyclic RGD peptide is reported to mediate delivery of NPs deep into the solid tumors,^33^, ^34^ we delivered FITC‐LTX/siR‐NPs into the mice by tail vein injection and investigated their penetration into the 4T1 tumors. As revealed in Figure S6, the fluorescence signal of the NPs without cRGD incorporation was confined to the tumor extracellular matrix, while the cRGD modification allowed the NPs to penetrate deeply into the tumor tissue.

To validate the antitumor efficacy of the LTX‐NPs, siR‐NPs, and LTX/siR‐NPs, we carried out the in vivo studies in a poorly immunogenic 4T1 breast tumor model by injecting PBS (control) and these NPs via the tail vein of the tumor‐bearing mice at a 3‐day interval for five treatments. All three NP formulations presented a potent effect on restraining the tumor progression, indicating via the significantly lower tumor volume and weight compared to the PBS treatment (Figure 3d–f). Notably, the LTX/siR‐NPs exhibited a superior antitumor activity than that of LTX‐NPs and siR‐NPs, which agrees with the in vitro observations that the combination of LTX‐315 and TGF‐β1 siRNA could amplify the tumor inhibition effect. Moreover, we conducted the hematoxylin and eosin (H&E) staining to observe the histological changes in the tumors harvested from treated mice. Figure 3g shows that the treatment with LTX/siR‐NPs induced necrotic and apoptotic condensations in the tumor tissues to the highest degree. In contrast, in the PBS‐treated group, no abnormality in tumor microstructure was observed.

**FIGURE 4:**
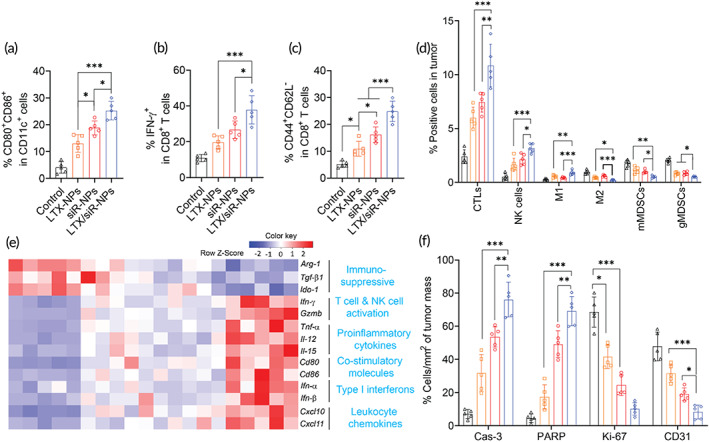
LTX/siR‐NPs re‐shaped the immune cell profile of tumor microenvironment. Flow cytometric analysis of (a) matured DC in tdLNs, (b) IFN‐γ produced by CD8^+^ T cells and (c) effector memory CD8^+^ T cells in the spleen, and (d) intratumoral profiles of immune cells of the mice treated with indicated formulations. (e) Heatmap visualization of gene expression in tumors collected from treated mice. (f) Cell percentages (%/mm^2^ of tumor mass) apoptosis markers (cleaved caspse‐3; cleaved PARP), tumor proliferation (Ki‐67), and angiogenesis marker (CD31) were detected by Immunohistochemical analysis. Data represented as mean ± SD (*n* = 5). One‐way ANOVA with Tukey's multiple comparison test, *p < 0.05; **p < 0.01; ***p < 0.001

Next, we investigated the initiation of the antitumor immune responses after LTX/siR‐NP treatment. As the LTX/siR‐NPs displayed a high ability to induce ICD and activation of DCs in vitro, we quantified the DC maturation markers (CD80 and CD86) in tumor‐draining lymph node (tdLNs) by flow cytometry. Figures 4a and S7a show that the treatment with the all hybrid NPs resulted in increased frequencies of CD11c^+^CD80^+^CD86^+^ cells in the tdLNs compared to the control group. The co‐treatment with LTX‐315 and TGF‐β1 siRNA in the same nanoplatform demonstrated more enhanced DC maturation than the single treatment. Following the capture of antigens and DAMP molecules released from dying tumor cells by DCs, the tumor‐specific effector T cells are primed and activated in response to the antigen‐presentation by the antigen‐captured, matured DCs.^35^, ^36^ Thus, we investigated the augment of the adaptive immune response in tumor‐bearing mice by ex vivo re‐stimulating lymphocytes isolated from the spleen of treated mice with PMA/ionomycin, followed by examining the intracellular levels of IFN‐γ, a hallmark cytokine of adaptive immunity that plays a critical role in the inhibition of viral, bacteria, and cancers,^37^, ^38^ in CD8^+^ T cells. As shown in Figures 4b and S7b, the treatment with LTX/siR‐NPs also presents the markedly increased percentages of IFN‐γ‐secreting CD8^+^ T cells compared to all other groups, demonstrating the promoted anticancer activity of cytotoxic CD8^+^ T cells in the LTX/siR‐NP‐treated mice. Given the importance of establishing the memory feature in cancer immunotherapy that provides the long‐term treatment benefit, we were further tested by examining the percentage of effector CD8^+^ T cells (CD3^+^CD8^+^CD44^+^CD62L^−^ cells) in splenocytes of treated mice. As shown in Figures 4c and S7c, the treatment with hybrid NPs resulted in a significantly higher effector CD8^+^ T cells than the PBS treatment.

Previous findings have revealed that the upregulation of TGF‐β1 in solid tumors enriches intratumoral collagen density, which is reported to mechanistically collaborate with TGF‐β‐related genes to decrease the population of cytotoxic T cells while promoting the exhausted CD8^+^ T cell phenotypes via LAIR1‐dependent manner, thereby promoting the resistance of tumors to immune checkpoint inhibitors.^39^ Thus, we performed the Masson Trichrome staining assay to assess whether the treatment with hybrid NPs delivering TGF‐β1 siRNA could decrease the collagen density within the tumor tissues. Figure S8 showed that significantly reduced tumor collagen deposition was detected in the tumor masses of mice treated with siR‐NPs and LTX/siR‐NPs. Next, we investigated the effect of the hybrid NP treatments on the intratumoral immune cell profiles. Compared with all other groups, the LTX/siR‐NP treatment induced significant increases in the infiltration of cytotoxic lymphocytes, including CD8^+^ T cells and NK1.1^+^ cells, and M_1_‐like tumor‐associated macrophages, while causing noticeably decreased numbers of immunosuppressive cells, including M_2_‐like tumor‐associated macrophages and monocytic and granulocytic myeloid‐derived suppressor cells (mMDSCs and gMDSCs, respectively; Figures 4d and S7d–g). To provide more insight into the underlying mechanisms by which the LTX/siR‐NPs turn the “cold” tumor into “hot”, we further evaluated the gene expression profiles within the tumor tissues of treated mice (Figures 4e and S9). As expected, LTX/siR‐NPs induced an inflamed and tumoricidal environment with significant increases of genes associated with T cell and NK cell activation (*Ifn‐γ*, *Grzmb*), co‐stimulatory molecules (*Cd80*, *Cd86*), proinflammatory cytokines (*Tnf‐α*, *Il‐12*, and *Il‐15*), leukocyte chemokines (*Cxcl10*, *Cxcl11*), and type I IFNs (*Ifn‐α*, *Ifn‐β*), whereas significantly downregulating expression of immunosuppressive genes, including *Arg‐1*, *Tgf‐β1*, and *Ido‐1*. Moreover, by immunohistochemical analysis, we detected that the LTX/siR‐NPs triggered the highest levels of the apoptosis markers (activated caspase‐3 and PARP) and reduced the marker of tumor proliferation (Ki‐67) and angiogenesis (CD31) in the tumors of treated mice much more than all other treated groups (Figures 4f, S10, and Table S2).

### 2.4 LTX/siR‐NPs synergized with NKG2A blockade therapy

**FIGURE 5:**
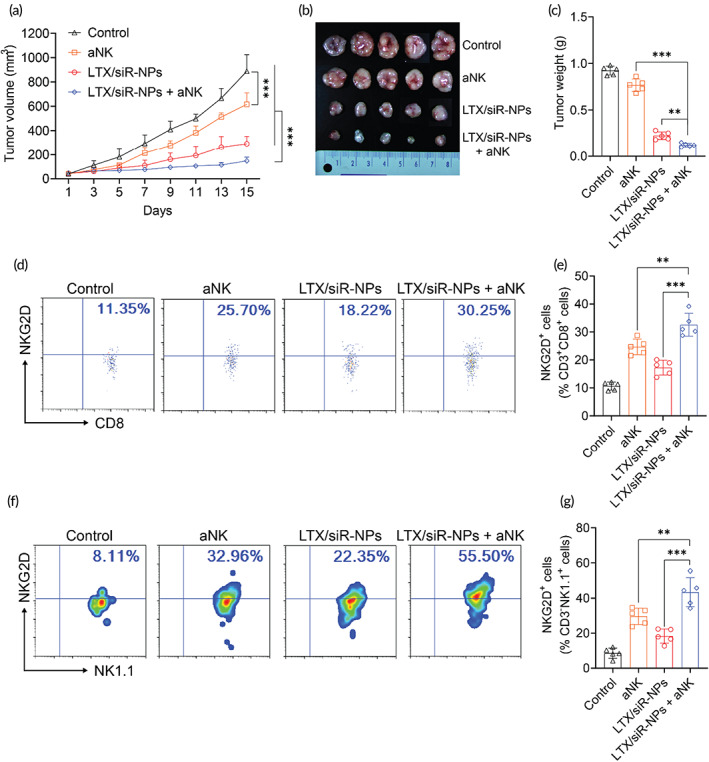
The synergic antitumor efficacy of LTX/siR‐NPs and NKG2A blockade. (a) Tumor volume of 4T1 tumor‐bearing mice treated with PBS (control), anti‐NKG2A antibody (aNK), LTX/siR‐NPs, and LTX/siR‐NPs + aNK with increasing time. (b) Images and (c) weight of 4T1 tumor isolated from treated mice. (d–g) Flow cytometric and data analysis of NKG2D expression in intratumoral CD8^+^ and NK cells. Data represented as mean ± SD (*n* = 5). Two‐way ANOVA (a) and one‐way ANOVA (c, e, g) with Tukey's multiple comparison test, *p < 0.05; **p < 0.01; ***p < 0.001

It is noted that approximately 50% and 5% of peripheral blood NK and T cells express the NKG2A receptor, respectively, at a steady state.^10^ Furthermore, it also reported the TGF‐β secreted by tumor cells noticeably induces expression of CD94 on their surface and NKG2A in CD8^+^ T cells and NK cells, thus resulting in the NKG2A‐mediated resistance to immunotherapies.^40^, ^41^ Convincing evidence suggests that by blocking NKG2A, the cytotoxic activity of NK cells and CD8^+^ T cells can be restored. As the LTX/siR‐NPs showed the ability to increase the intratumoral number of CD8^+^ T cells and NK cells, we established a combination therapy of LTX/siR‐NPs and anti‐NKG2A antibody (aNK) for enhancing the activity of these lymphocytes against the tumor. As shown in Figure 5a–c, the single treatment of aNK could slightly slow down the tumor growth, while the combination of LTX/siR‐NPs and anti‐NKG2A presented an almost complete tumor suppression. Furthermore, this combination therapy showed to prolong the survival rate of the treated mice compared to the monotherapies (Figure S11a). Consistently, the flow cytometric analysis (Figure 5d–g) also showed that the synergistically enhanced cytotoxic function of both CD8^+^ T cells and NK cells was achieved when combining LTX/siR‐NPs with anti‐NKG2A therapy, indicating via the upregulation of NKG2D, the activating receptor functioning in triggering the cytotoxicity against the transformed and stress cells, including tumor cells.^5^, ^42^ Moreover, we observed no significant changes in body weight of mice following all the treatments (Figure S11b), showing the safety of this combination therapy. These results provide propelling evidence for the potential of LTX/siR‐NPs in the immune checkpoint blockade‐based combination regimen.

## 3 CONCLUSION

In this study, we designed and successfully prepared the hybrid LTX/siR‐NPs for promoting the antitumor immune responses in the “cold” tumors which are associated with the downregulation of tumor‐associated antigens, the poor tumor‐infiltrated lymphocytes, and the cancer cell‐intrinsic and TME resistance mechanisms. The in vitro results showed that the LTX/siR‐NPs could effectively inhibit TGF‐β1 expression and convert the nonimmunologically silent tumor cell death into an immunogenic way accompanied by the release of tumor‐associated antigens and danger signals for the DC activation. More importantly, the LTX/siR‐NPs effectively accumulated and penetrated deeply into the tumors upon intravenous injection, creating an immunologically active tumor with significantly elevated levels of cytokines and chemokines involved in T cell and NK cell recruitment and the disruption of immunosuppressive components. When combining the LTX/siR‐NP treatment with NKG2A blockade therapy, the cytotoxic function of intratumoral CD8^+^ T cells and NK cells was markedly enhanced, leading to a superior antitumor effect. Taken together, our work suggests the potential of the LTX/siR‐NPs for inflaming the “cold” tumor for potentiating the efficacy of cancer immunotherapy.

## 4 EXPERIMENTAL SECTION

### 4.1 Materials

LTX‐315 (sequence: K‐K‐W‐W‐K‐K‐W‐Dip‐K‐NH_2_), FITC‐LTX‐315 (N‐ter modification), and Cyclo(‐RGDfC) peptides were synthesized and provided by GeneCust (Rue de Chalmont, Boynes, France). Murine TGF‐β1 siRNA (Forward, GCAACAACGCCAUCUAUGA; Reverse, UCAUAGAUGGCGUUGUUGC) and Cyanine 5.5 conjugated TGF‐β1 siRNA were synthesized and provided by Bioneer (Daedeok‐gu, Daejeon, South Korea). Poly(d,l‐lactide‐co‐glycolide) (Resomer® RG 504, Mw 38,000–54,000) was purchased from Sigma Aldrich (St. Louis, MO). DSPE‐PEG(2000) was procured from Nanocs (Fifth Avenue, NY). DSPE‐PEG(2000) Maleimide was obtained from Avanti Polar Lipids (Industrial Park Drive Alabaster, Alabama). InVivoMAb anti‐mouse NKG2A/C/E antibody was received from BioXCell (West Lebanon, NH). All fluorophore‐modified antibodies used in flow cytometry were purchased from Biolegend (San Diego, CA).

### 4.2 Cell culture

4T1 cells (ATCC® CRL‐2539™) were purchased from American Type Culture Collection (Manassas, VA) and cultured in RPMI 1640 supplemented with 10% FBS, and 100 IU/ml penicillin/streptomycin.

BMDCs were derived from total bone marrow cells harvested from the femurs of 6‐ to 8‐week‐old C57BL/6 mice as previously reported.^2^ Briefly, the bone marrow cells were collected from femur and tibia bones. The red blood cells (RBCs) were removed by using 1X RBC Lysis Buffer (Biolegend, San Diego, CA) after the bone marrow cells had passed through 70 μm cell strainer (Falcon®, Corning Costar, NY). The obtained cells were placed in 150 mm Petri dish at a density of 10^6^ cells/dish in the presence of the recombinant mouse granulocyte‐macrophage colony‐stimulating factor (GM‐CSF; 20 ng/ml; Biolegend, San Diego, CA). The cultured media was changed every 3 days and the BMDCs were harvested on Day 8. The fully differentiated BMDCs were checked by the expression of CD11c (Figure S5a) and maintained in RPMI‐1640 supplemented with 10% FBS, 100 IU/ml penicillin/streptomycin, and 20 ng/ml recombinant mouse GM‐CSF.

### 4.3 Animals

All experiments in female Balb/c mice were approved and followed the guidance of the Institutional Authority for Laboratory Animal Care and Handling, Yeungnam University, Gyeongsan, Republic of Korea.

### 4.4 DSPE‐PEG‐cRGD synthesis

The conjugations of cRGD to DSPE‐PEG were performed as the published method.^43^ Briefly, DSPE‐PEG‐Mal was dissolved in 0.1 M PBS (pH 7.2, 0.15 M NaCl, 2 mM EDTA) containing triethylamine (2 equiv.), cRGD (2 equiv.) was then added to the reaction solution with stirring. The reaction solution was stirred for 12 h at room temperature in the dark. Then, the reaction solution was dialyzed against ultrapure water using a 3000 Da cut‐off dialysis membrane for 24 h to remove the free material. Finally, the DSPE‐PEG‐cRGD was obtained by lyophilizing and stored at −20°C for further studies. The product structure was confirmed by ^1^H‐NMR spectroscopy using CDCl_3_ as the solvent.

### 4.5 Preparation of LTX/siR‐NPs

The LTX/siR‐NPs were prepared using the double‐emulsion method.^30^, ^44^ Briefly, 400 μl of RNase‐free water containing 2.5 mg/ml LTX‐315 and 5 μM TGF‐β1 siRNA (~66.5 μg/ml) was emulsified in 2 ml of dichloromethane (DCM) dissolved 5 mg of DSPE‐mPEG, 5 mg of DSPE‐PEG‐cRGD, and 20 mg of PLGA using a probe sonication at 30% power for 1 min on ice. Subsequently, 8 ml of RNase‐free water was added to the primary emulsion and the sample was further subjected to the probe sonication as before. The DCM was evaporated using a rotary evaporator. The hybrid NPs were next harvested by centrifugation at 10,000 *g* for 15 min, and washed with DI water. All supernatants were saved to determine the amount of untrapped LTX‐315 and siRNA. The obtained NPs were resuspended in 50 mg/ml of sucrose, freeze‐dried, and kept in −20°C for further uses. The NPs encapsulating LTX‐315, TGF‐β1 siRNA (denoted as LTX‐NPs and siR‐NPs, respectively) were prepared in the same method as the LTX/siR‐NPs.

### 4.6 Nanoparticle characterization

Size and zeta potential of the hybrid NPs were measured using a DLS instrument (Zetasizer Nano, Malvern, UK). Morphology of NPs was examined by staining them with 2% phosphotungstic acid solution before mounting on carbon‐coated copper grids and observed using TEM (CM 200 UT; Philips, Andover, MA). The successful entrapment of LTX‐315 and siRNA into the hybrid NPs were confirmed by recording the UV–VIS spectra of Cy5.5‐siRNA, DSPE‐PEG‐cRGD, LTX‐315, and LTX/Cy5.5‐siR‐NPs using a U‐2800 UV–VIS spectroscopy (Hitachi, Tokyo, Japan). The changes in hydrodynamic size and surface charge of LTX/siR‐NPs in different pH conditions were examined by soaking them in phosphate buffer (pH 7.4 and 6.5) and acetate buffer pH 5.5 for 2 h, followed by subjecting them to DLS for measuring the size and zeta potential. The complexation of the siRNA and LTX‐315 peptide was characterized using agarose electrophoresis. Briefly, LTX‐315 and siRNA in RNase‐free water were mixed at different N/P ratios and incubated at room temperature for 30 min. After that, 6X DNA loading buffer (Thermo Fisher Scientific, Waltham, MA) was added to the mixtures, followed by loading them into 2% (w/v) agarose gel stained with Gelred (Biotium, Hayward, CA) in Tris‐borate EDTA buffer (Promega Corporation, Fitchburg, WI). The electrophoresis was performed at 100 V for 15 min. The agarose gel image was captured using a UV illuminator. The entrapment efficiency of the LTX‐315 and siRNA was indirectly determined by calculating the amount of untrapped LTX‐315 and siRNA in the supernatants collected from the NP purification steps. The concentration of LTX‐315 was measured using an HPLC method with a gradient elute mode (Table S1) using a reversed‐phase Inertsil ODS‐3 column (5 μm, 4.6 × 150 mm; GL Sciences Inc., Rolling Hills Estates, CA); injection volume was 5 μl; and UV detector at 280 nm. The siRNA amount was determined using a QuantiFluor® RNA System Kit (Promega, Madison, WI) according to the manufacturer's instructions. The release patterns of LTX‐315 and siRNA from the LTX/siR‐NPs were investigated in different pH environments. Briefly, the LTX/siR‐NPs were incubated in 1 ml of acetate buffer pH 5.5, phosphate buffer pH 6.5, and pH 7.4 under a shaking speed of 100 rpm. At different time points, the NP suspension was centrifuged at 10,000 *g* for 15 min, and 0.5 ml of supernatants were collected for the measurement of LTX‐315 and siRNA concentration, and 0.5 ml of the fresh buffers were added to resuspend the precipitates for further incubation. The siRNA amount was measured using a Picogreen kit (Thermo Fisher Scientific) following the manufacturer's protocol, while the LTX‐315 concentration was quantified using the HPLC method described above. The stability of LTX/siR‐NPs in serum was tested by immersing the NPs in PBS buffer pH 7.4 supplemented with 10% FBS at 37°C. At indicated time intervals, their size and PDI were determined by the DLS instrument. To measure the adsorption of serum proteins on the LTX/siR‐NP surface, the NP suspension was collected and centrifuged at 10,000 *g* for 15 min, and the supernatants were saved for calculating the nonadsorbed protein concentration using a BCA protein assay kit (Thermo Fisher Scientific). The amount of adsorbed proteins was calculated by subtracting the total serum protein amount from the nonadsorbed protein amount.

### 4.7 Cellular uptake studies

The internalization of the hybrid NPs into the 4T1 cells was investigated by flow cytometry, and confocal laser scanning microscopy (CLSM), in which the FITC‐conjugated LTX‐315 (FITC‐LTX‐315) was loaded into the NPs modified with cRGD (targeted NPs) or nonmodified NPs (nontargeted NPs). For flow cytometric analysis, the 4T1 cells were seeded into 6‐well plates at a density of 2 × 10^5^ cells/well overnight. Thereafter, the cells were treated with either targeted or nontargeted NPs at a FITC‐LTX‐315 concentration of 5 μg/ml for 1 h. After washing thrice with PBS, the cells were harvested and analyzed their intracellular fluorescence intensity by FACSVerse (BD Biosciences, San Jose, CA).

For the CLSM examination, the cells were cultured in 12‐well plates covered by microscope coverslips (Thomas Scientific, Swedesboro, NJ) at a density of 2 × 10^5^ cells/well. On the next day, the cells were incubated with either targeted or nontargeted NPs at a FITC‐LTX‐315 concentration of 5 μg/ml for 1 h, followed by the addition of 2 nM Lysotracker Red (Thermo Fisher Scientific) and incubated for a further 10 min. Next, the cells were washed thrice with PBS and fixed with 4% paraformaldehyde, and incubated with 10 μg/ml Hoechst 33342 (Thermo Fisher Scientific). Finally, the stained cells were mounted on a glass slide and observed under K1‐Fluo CLSM (Nanoscope, Daejeon, South Korea).

For observation of endosomal escape of the siRNA, the FAM‐labeled siRNA (FAM‐siR) was loaded into the hybrid NPs and incubated with the 4T1 cells seeded into 12‐well coverslip plates for 8 h, followed by the addition of 2 nM Lysotracker Red and 10 μg/ml Hoechst 33342. The coverslips containing the stained cells were then examined as described above.

### 4.8 Western blotting analysis

The 4T1 cells were grown in 6‐well plates (2 × 10^5^ cells/well) and incubated with PBS (control), blank NPs, LTX‐315, LTX‐NPs, siR‐NPs, and LTX/siR‐NPs at equivalent concentrations of LTX‐315 and TGF‐β1 siRNA was 10 μg/ml and 50 nM, respectively, for 48 h. Next, the cells were harvested, and extracted total proteins using M‐PER™ Mammalian Protein Extraction Reagent (Thermo Fisher Scientific) supplemented with 5% (v/v) EDTA‐free proteinase inhibitor cocktail (Roche Diagnostics, Risch‐Rotkreuz, Switzerland). The cellular protein concentration was measured using a BCA Protein Assay Kit (Thermo Fisher Scientific). Then, 20 μg of proteins were mixed with 5X Lane Marker Reducing Sample Buffer (Thermo Fisher Scientific), boiled for 5 min, loaded into 10% sodium dodecyl sulfate‐polyacrylamide (SDS‐PAGE) gel, and electrophoresed at 60 V for 2 h using a Power Supply (PS300‐B, Hoefer Inc., Holliston, MA). The separated proteins were then transferred to the Immobilon® PVDF membranes (Merck Millipore, Burlington, MA) in a Western Blot Transfer Buffer (ThermoFisher Scientific). After that, the membranes were blocked with 5% Bovine Serum Albumin (BSA) dissolved in Tris‐Buffered Saline supplemented with 0.1% Tween‐20 (TBST) for 1 h prior to incubating with rabbit anti‐mouse TGF‐β1 (Abcam, Cambridge, UK) or mouse anti‐mouse GAPDH (ab8245, Abcam, Cambridge, UK) antibody diluted in 5% BSA solution at 4°C overnight. The membranes were next washed with TBST solution and incubated with HRP‐linked anti‐rabbit secondary IgG for detection of TGF‐β1 and HRP‐linked anti‐mouse secondary IgG for detection of GAPDH. Finally, the protein signals on the membrane were detected and imaged by a Kodak imaging instrument (Kodak, Rochester, NY) after 15‐min incubation with Supersiganal West Femto kit (Thermo Fisher).

### 4.9 Evaluation of immunogenic cell death effect

The toxicity of the LTX‐315 and different hybrid NP formulations on 4T1 cells was tested by MTS assay. Briefly, 1 × 10^4^ 4T1 cells were grown in 96‐well plates and treated with various dilutions of LTX‐315, blank NPs, LTX‐NPs, siR‐NPs, and LTX/siR‐NPs for 48 h. Next, the viability of treated cells was determined using MTS reagent (Promega, Madison, WI) according to the supplier's protocol. The absorbance (OD) at 490 nm was measured using a Multiskan microplate reader (Thermo Fisher Scientific). The tumor cell viability was calculated following the formula:

$$\text{Viability}\;\left(\%\right)=\frac{\mathrm{OD}\;\left(\text{sample}\right)-\mathrm{OD}\;\left(\text{blank}\right)}{\mathrm{OD}\;\left(\text{control}\right)-\mathrm{OD}\;\left(\text{blank}\right)}\times 100$$

To analyze the membrane expression of calreticulin and release of ATP from 4T1 cells after treatment, the tumor cells were seeded into 6‐well plates at a density of 2 × 10^5^ cells/well and treated with LTX‐315, blank NPs, LTX‐NPs, siR‐NPs, and LTX/siR‐NPs at equivalent LTX‐315 and siRNA concentration of 10 μg/ml and 50 nM, respectively for 24 h. Then, the conditioned medium was collected for measuring the ATP levels, and cells were incubated with rabbit anti‐mouse calreticulin antibody (1 μg/ml; ab2907, Abcam) for 30 min on ice, followed by incubating with Goat anti‐Rabbit IgG secondary antibody, DyLight 488 (1 μg/ml; Thermo Fisher Scientific), for additional 30 min in the dark. Finally, the cells were subjected to flow cytometry for analysis of the surface CRT fluorescence intensity. The ATP concentrations in the medium of treated cells were determined using the CellTiter‐Glo kit (Promega) following the company's instructions.

For the detection of intracellular HMGB1 and ATP levels, the 4T1 cells were seeded onto 12‐well plates covered by microscope coverslips and incubated with 5 μM quinacrine (Sigma Aldrich) and 10 μg/ml Hoechst 33342 for 30 min. After washing with PBS, the nuclear HMGB1 was stained with PE anti‐mouse HMGB1 antibody using intranuclear staining kit (Cat. 00‐5521‐00, Thermo Fisher Scientific) following the manufacturer's protocol. The coverslips containing the stained cells were mounted on a glass slide and observed under K1‐Fluo CLSM.

To elucidate that DCs can be activated by immunogenic cell death effect of tumor cells, 4T1 cells were cultured in 12‐well plates at the density of 10^5^ cells/well and pretreated with free LTX‐315, blank NPs, LTX‐NPs, siR‐NPs, or LTX/siR‐NPs (10 μg/ml of LTX‐315 and 50 nM of siRNA basis) for 24 h. Then, the cell culture media was collected and centrifuged at 10,000 *g* for 15 min to completely remove the remaining nanoparticles as well as the cell debris and obtain the supernatants. After that, the supernatants were added to the BMDCs and incubated for 24 h. Finally, the treated BMDCs were harvested to analyze the expressions of surface markers and the change in the gene expression of pro‐inflammatory cytokines. For detecting surface markers, the collected BMDCs were incubated with anti‐mouse CD16/32 for 30 min at 4°C and stained with APC anti‐mouse CD11c, Per/CP anti‐mouse MHC‐II, FITC anti‐mouse CD80, and APC/Cy7 anti‐mouse CD86 antibodies. The expression of these markers on the DC membrane was observed by flow cytometry. To evaluate the expressions of pro‐inflammatory cytokines, total RNA was extracted from treated BMDCs. The expression levels of interesting RNAs were then determined by an RT‐PCR method.

### 4.10 Pharmacokinetic and biodistribution studies

For the pharmacokinetic study, 8 weeks old BALB/c mice were injected with FITC‐LTX‐315 or FITC‐LTX/siR‐NPs via the tail vein. At indicated time points, the mice were euthanized and the blood was taken from the heart. Sodium heparin was added to the blood samples at a concentration of 15 IU/ml. The fluorescence intensity of FITC‐LTX‐315 in the serum was measured using Tecan Infinite F200 Fluorescence Microplate Reader (Tecan, Seestrasse, Männedorf, Switzerland) at an excitation and emission wavelength of 485 and 535 nm, respectively.

For the biodistribution study, the hybrid NPs modified with cRGD (targeted NPs) or nonmodified NPs (nontargeted NPs) co‐entrapping LTX‐315 and Cyanine 5.5‐labeled siRNA (Cy5.5‐siR) were intravenously injected to the 4T1 tumor‐bearing BALB/c mice. At predetermined time points, the Cy5.5‐siR signal in the mice was captured using FOBI imaging instrument (NeoScience; Suwon, South Korea). After 24 h, the tumors and major organs were harvested for measuring the fluorescence intensity.

For determining how deep the NPs with or without the surface modification, 4T1 tumor‐bearing mice were injected with FITC‐LTX‐315 loaded NPs. After 24 h, treated mice were sacrificed to obtain the tumors. The tumors were then sliced and stained with DAPI and the nanoparticle penetrations into the tumor mass were detected using Nikon Elipse Ti Fluorescence Microscopy.

### 4.11 In vivo therapeutic efficacy of LTX/siR‐NPs

To establish the 4T1 syngeneic murine model, 6 weeks old female BALB/c mice were inoculated at the right flank with 1 × 10^6^ 4T1 cells resuspended in 100 μl serum‐free RPMI media. When the tumor volume reached ~80 mm^3^, the mice were randomly divided into four groups (*n* = 5, denoted as Day 1) and intravenously injected with PBS (control), LTX‐NPs, siR‐NPs, or LTX/siR‐NPs at 20 mg/kg LTX‐315 and 1 mg/kg siRNA on the basis at day 1, 4, 7, 10, and 13. During the study period, the length and width of tumors were measured using a digital caliper (CD‐15CPX, Mitutoyo, Tokyo, Japan). The tumor volume (mm^3^) was calculated using the formula: *V* = 0.5 × length × width^2^. On Day 15, the mice were euthanized, and the tumor, inguinal lymph node, and spleen were harvested for further examinations.

For the combination study of LTX/siR‐NPs and NKG2A blockade therapy, the 4T1 tumor‐bearing mice were randomly divided into four groups corresponding to the treatment with PBS (control), LTX/siR‐NPs (20 mg/kg LTX‐315 and 1 mg/kg siRNA), anti‐mouse NKG2A/C/E antibody (aNK, 10 mg/kg), and LTX/siR‐NPs + aNK (20 mg/kg LTX‐315, 1 mg/kg siRNA, and 10 mg/kg antibody), in which the LTX/siR‐NPs were intravenously injected on Day 1, 4, 7, 10, and 13, and the antibody was intraperitoneally injected on Day 2, 5, 8, 11 and 14. On Day 15, the tumors were collected for flow cytometric analysis.

### 4.12 Gene expression analysis following LTX/siR‐NP treatment

The tumors collected from mice treated with PBS, LTX‐NPs, siR‐NPs, and LTX‐siR‐NPs were cut into small pieces and incubated in RPMI supplemented with 10 U/ml collagenase I, 400 U/ml collagenase IV, and 30 U/mL DNAse I (ThermoFisher Scientific) at 37°C for 1 h. The tumor pieces were then homogenized using a syringe plunger and filtered through 70 μm cell strainers (Sigma‐Aldrich). The erythrocytes in the tumor cells were then lysed using RBC lysis buffer (BioLegend). The RBC‐depleted single cells were then lysed using TRIzol reagent (Invitrogen, Carlsbad, CA) for the extraction of total RNA following the supplier's protocol. The extracted RNA (2 μg) was subjected to a reverse transcription reaction to synthesize cDNA using GoScript Reverse Transcription kit (Promega). A mixture of cDNA (2 μl), 2X ABsolute qPCR SYBR Green Mix (10 μl, Thermo Fisher Scientific, Waltham, MA), forward and reverse primers (2 μM, 1.5 μl each), and nuclease‐free water (5 μl) was then added into LightCycler Capillaries (Roche Diagnostics, Risch‐Rotkreuz, Switzerland), followed by an amplification reaction using a LightCycler Real‐Time PCR instrument (Roche Diagnostics). The mRNA expression fold‐changes were calculated by the delta–delta C_p_ method using GAPDH as housekeeping gene following previous studies.^45^ The primer sequences were listed in Table S3.

### 4.13 Ex vivo re‐stimulating lymphocytes

The splenocytes were isolated from harvested spleens. Briefly, the spleens were ground by 3‐ml syringe plunger and then passed through 70 μm cell strainer (Sigma‐Aldrich). The RBCs were lysed using 1× RBC lysis buffer (Biolegend) for 15 min at room temperature. After that, isolated cells were cultured in 96‐well plates and stimulated with phorbol 12‐myristate 13‐acetate (PMA) and ionomycin for 24 h. The intracellular IFN‐γ cytokines were stained by eBioscience™ Intracellular kit (Cat. 88‐8824‐00, Thermo Fisher) and detected by flow cytometry.

### 4.14 Flow cytometry

The RBC‐depleted single cell suspensions were prepared as mentioned method. The isolated cells were then incubated with anti‐mouse CD16/32 antibodies (clone 93, Biolegend, San Diego, CA) on ice, followed by staining with several fluorescence‐conjugated anti‐mouse antibodies (Table S4) by the manufacturer's protocol. Finally, fluorescent signals were detected by flow cytometry, and the obtained data were analyzed by FlowJo VX software.

### 4.15 Histological and immunohistochemical analysis

Tumors and major organs harvested from tumor‐bearing mice on the final day of treatments were fixed in 10% neutral‐buffered formalin. After embedding in paraffin, 4 μm‐thickness sections from those tissues were stained with hematoxylin and eosin (H&E) or used for immunohistochemical analysis. For immunohistochemistry, the sections were deparaffinized and blocked with normal horse serum blocking solution for 1 h followed by treatment with primary antisera of cleaved caspase‐3; cleaved PARP; Ki‐67; CD31; CD8; or NK1.1 overnight at 4°C in the humidity chamber. After that, these samples were incubated with biotinylated universal secondary antibody and ABC reagents for 1 h at room temperature in a humidity chamber. The areas occupied by positive cells (%/mm^2^ of tumor mass) and the mean numbers of positive cells (cells/mm^2^ of tumor mass) were measured by a histological camera system and automated image analyzer as in the previous report.^46^

### 4.16 Masson trichrome staining assay

Tumor sections with the thickness of 4 μm were prepared as the above method. The collagen densities in the tumor mass were detected using NovaUltr™ Masson Trichrome Stain Kit (IW‐3006, IHC World, MD21042) following the manufacturer's protocol. The photographs were acquired by Nikon Elipse Ti Fluorescence Microscopy and quantification of collagen densities were measured using ImageJ software.

### 4.17 Statistical analysis

The data were shown as mean ± SD. For multiple comparisons, one‐way ANOVA with Tukey's test was performed. For individual comparisons, a two‐tailed Student's *t*‐test was used. The statistical significances were represented by the mark “*” if *p* < 0.05 and “**, ***” if *p* < 0.01 and 0.001, respectively.

## AUTHOR CONTRIBUTIONS

**Cao Dai Phung:** Conceptualization (equal); data curation (equal); methodology (equal); writing – original draft (equal). **Bao Loc Nguyen:** Conceptualization (equal); data curation (equal); formal analysis (equal); methodology (equal); writing – original draft (equal). **Jee‐Heon Jeong:** Data curation (equal); formal analysis (equal); writing – review and editing (equal). **Jae‐Hoon Chang:** Formal analysis (equal); investigation (equal); resources (equal). **Sung Giu Jin:** Formal analysis (equal); investigation (equal); resources (equal). **Han‐Gon Choi:** Formal analysis (equal); investigation (equal); resources (equal). **Sae Kwang Ku:** Data curation (equal); formal analysis (equal); investigation (equal); methodology (equal). **Jong Oh Kim:** Conceptualization (equal); funding acquisition (equal); supervision (equal); writing – review and editing (equal).

## CONFLICT OF INTEREST

The authors have no conflict of interest to declare.

## Supporting information


**Appendix S1** Supporting Information.

## Data Availability

All data needed to evaluate the conclusions in the paper are present in the paper and/or the Supplementary Materials. Additional data related to this paper may be requested from the authors.
