# Supplementary material for: Shaping the “hot” immunogenic tumor microenvironment by nanoparticles co‐delivering oncolytic peptide and TGF‐β1 siRNA for boosting checkpoint blockade therapy
Source: Bioeng Transl Med. 2022 Aug 11;8(5):e10392. doi: 10.1002/btm2.10392 (PMC10487304; doi:10.1002/btm2.10392)
Supplement: Supplementary file 1 — Appendix S1 Supporting Information. [file BTM2-8-e10392-s001.docx]

**Supplementary information**

**Shaping the "hot" immunogenic tumor microenvironment by nanoparticles co-delivering oncolytic peptide and TGF-β1 siRNA for boosting checkpoint blockade therapy**

Cao Dai Phung^1§^, Bao Loc Nguyen^1§^, Jee-Heon Jeong^2^, Jae-Hoon Chang^1^, Sung Giu Jin^3^, Han-Gon Choi^4^, Sae Kwang Ku^5^, Jong Oh Kim^1,*^

^1^College of Pharmacy, Yeungnam University, Gyeongsan 38541, Republic of Korea

^2^Department of Precision Medicine, School of Medicine, Sungkyunkwan University, Suwon 16419, Republic of Korea

^3^Department of Pharmaceutical Engineering, Dankook University, 119 Dandae-ro, Dongnam-gu, Cheonan 31116, Republic of Korea

^4^College of Pharmacy & Institute of Pharmaceutical Science and Technology, Hanyang University, 55 Hanyangdaehak-ro, Sangnok-gu, Ansan 15588, Republic of Korea

^5^College of Korean Medicine, Daegu Haany University, Gyeongsan 38610, Republic of Korea

^*^Corresponding author:

Prof. Jong Oh Kim, Ph.D

Tel: +82-53-810-2813, Fax: +82-53-810-4654, E-mail: [jongohkim@yu.ac.kr](mailto:jongohkim@yu.ac.kr)

^§^ Cao Dai Phung and Bao Loc Nguyen contributed equally to this work.


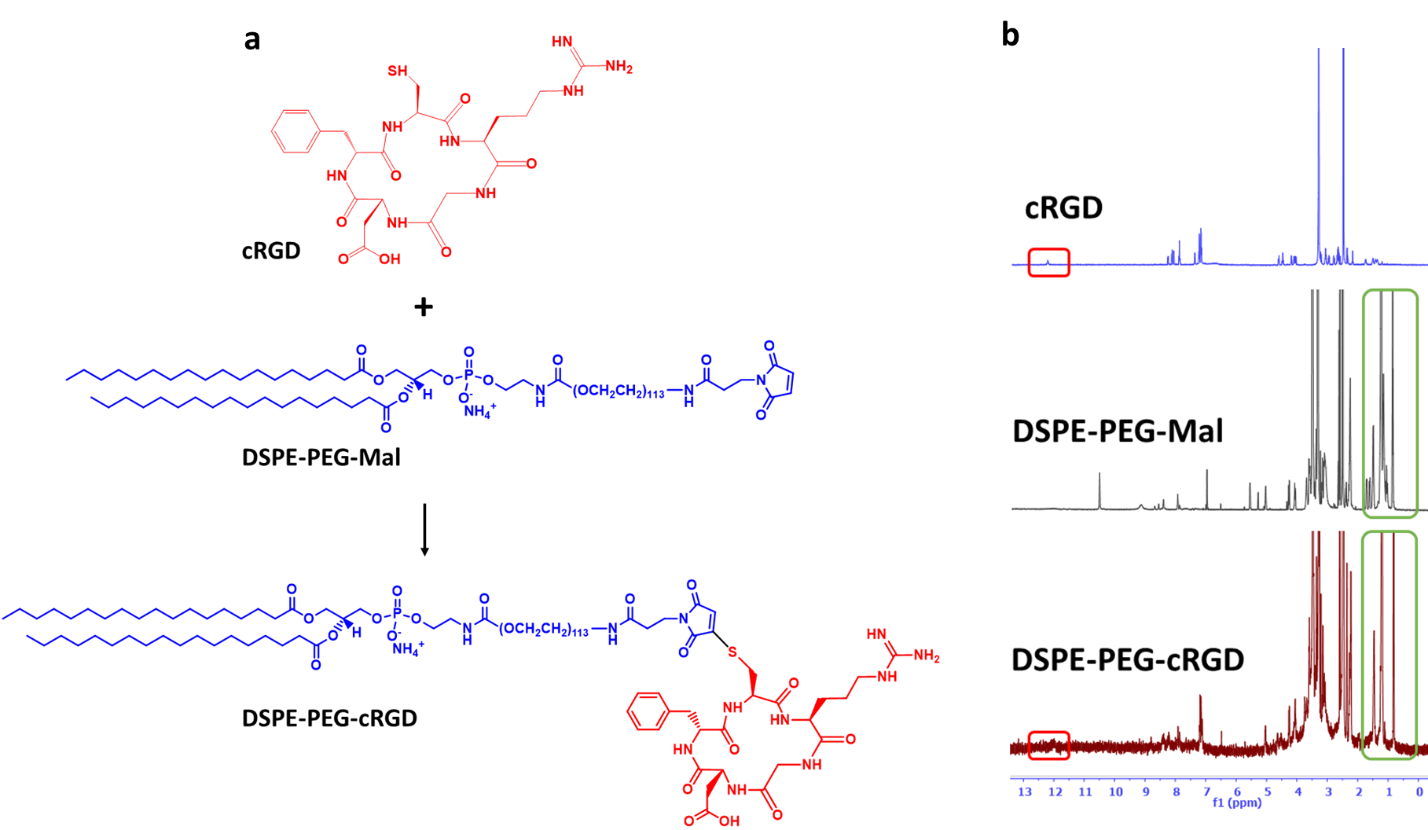


**Figure S1.** Synthesis and characterization of DESPE-PEG-cRGD**. (a)** Schematic of DSPE-PEG-cRGD synthesis and **(b)** ^1^H-NMR spectra of cRGD, DSPE-PEG-Maleimide, and DSPE-PEG-cRGD.


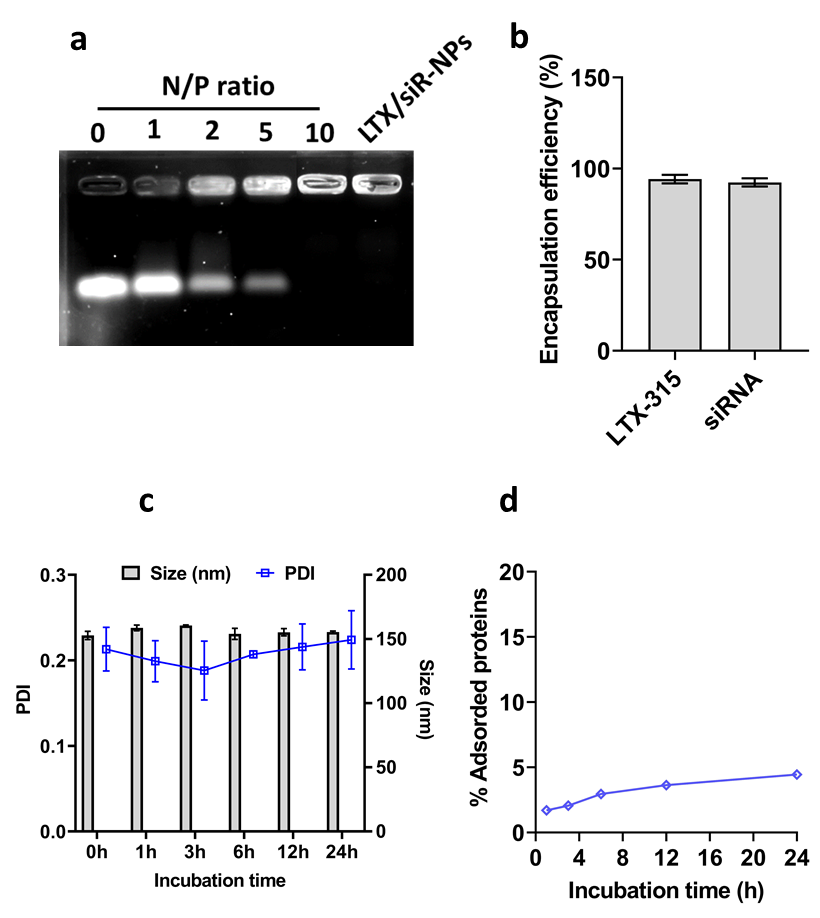


**Figure S2.** Characterization of LTX/siR-NPs. **(a)** Agarose gel electrophoresis analysis of LTX-315/siRNA complexation at various N/P ratios and LTX/siR-NPs at the presence of 25 pmole siRNA. **(b)** Encapsulation efficiencies of LTX-315 and siRNA in hybrid NPs. **(c)** Stability of LTX/siR-NPs demonstrated by measuring nanoparticle size and PDI value. **(d)** The absorption of serum protein onto the surface of LTX/siR-NPs at different incubation time.


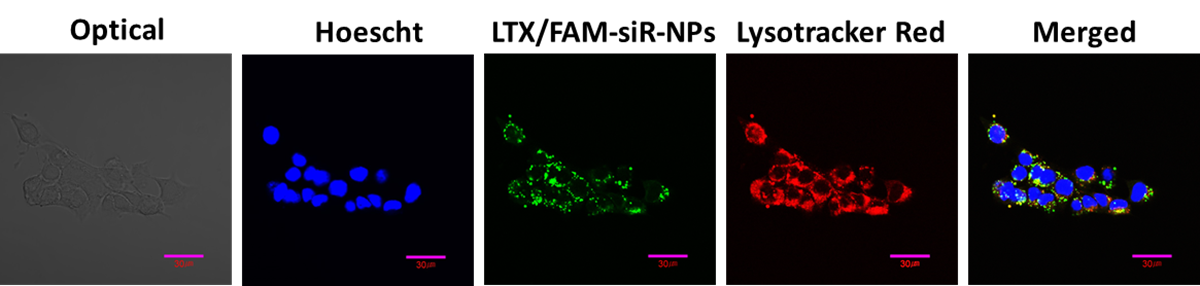


**Figure S3.** Confocal laser scanning microscopic observation for the dissociation of siRNA from endolysosomes in the 4T1 cancer cells after treatment with LTX/FAM-siR-NPs. Scale bar: 30 μm. Blue: Hoechst, Red: Lysotracker Red, Green: FAM-siRNA.


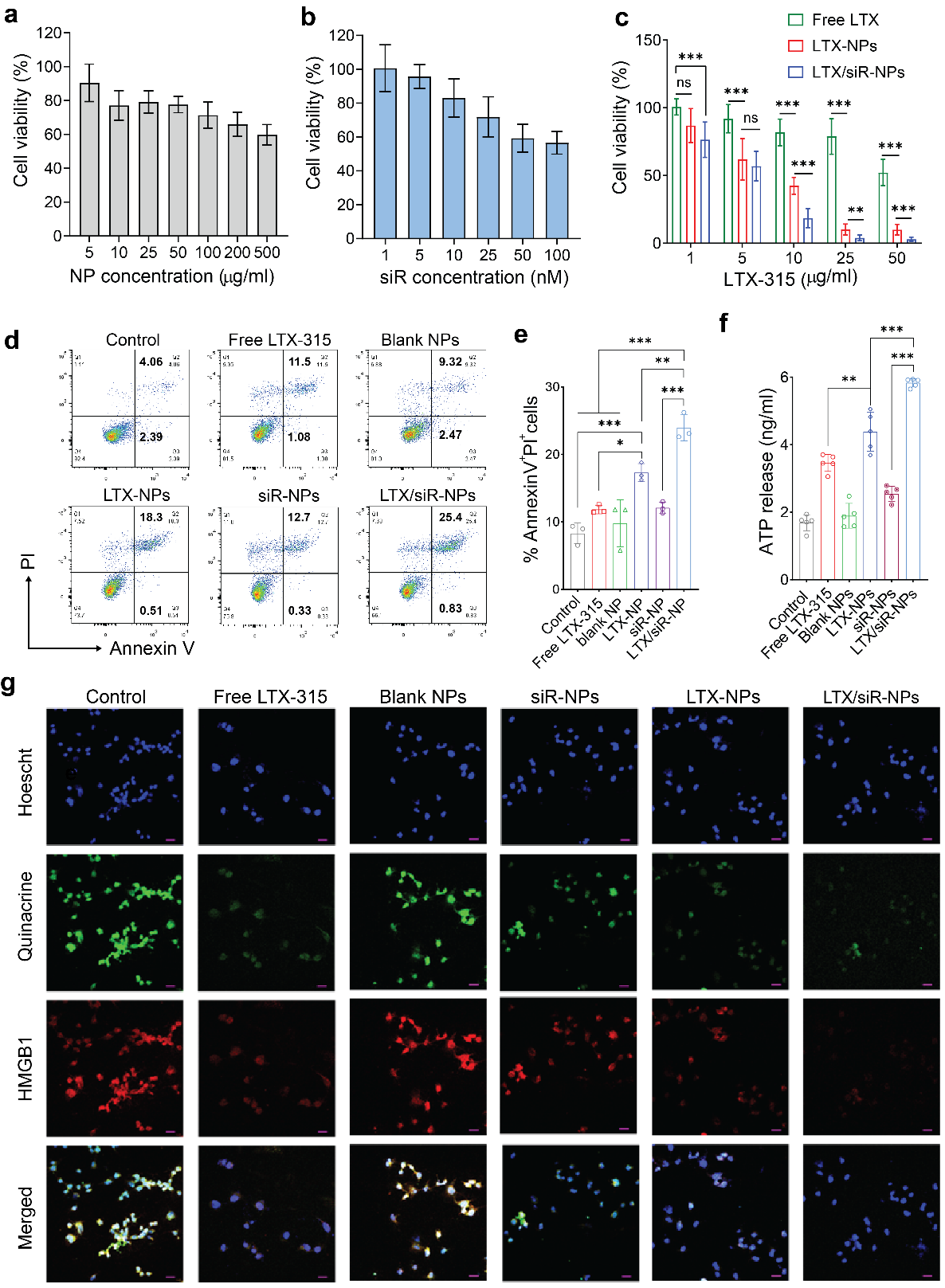


**Figure S4.** Anticancer effect of LTX-315-contained NPs on 4T1 cells. (**a-c**) Viability of 4T1 cells after treatment with free LTX-315 and various NP formulations. **(d-e)** The apoptosis of cancer cells treated with different formulations were detected by Annexin V/PI double staining. **(f)** The extracellular release of ATP from cancer cells treated with free LTX-315 and drug loaded nanoparticles. Data were presented as mean ± SD (n=3). **, p<0.01; ***, p<0.001. (**g**) Intracellular level of ATP and HMGB1 in treated 4T1 detected by CLMS**.** Scale bar: 20 µm.


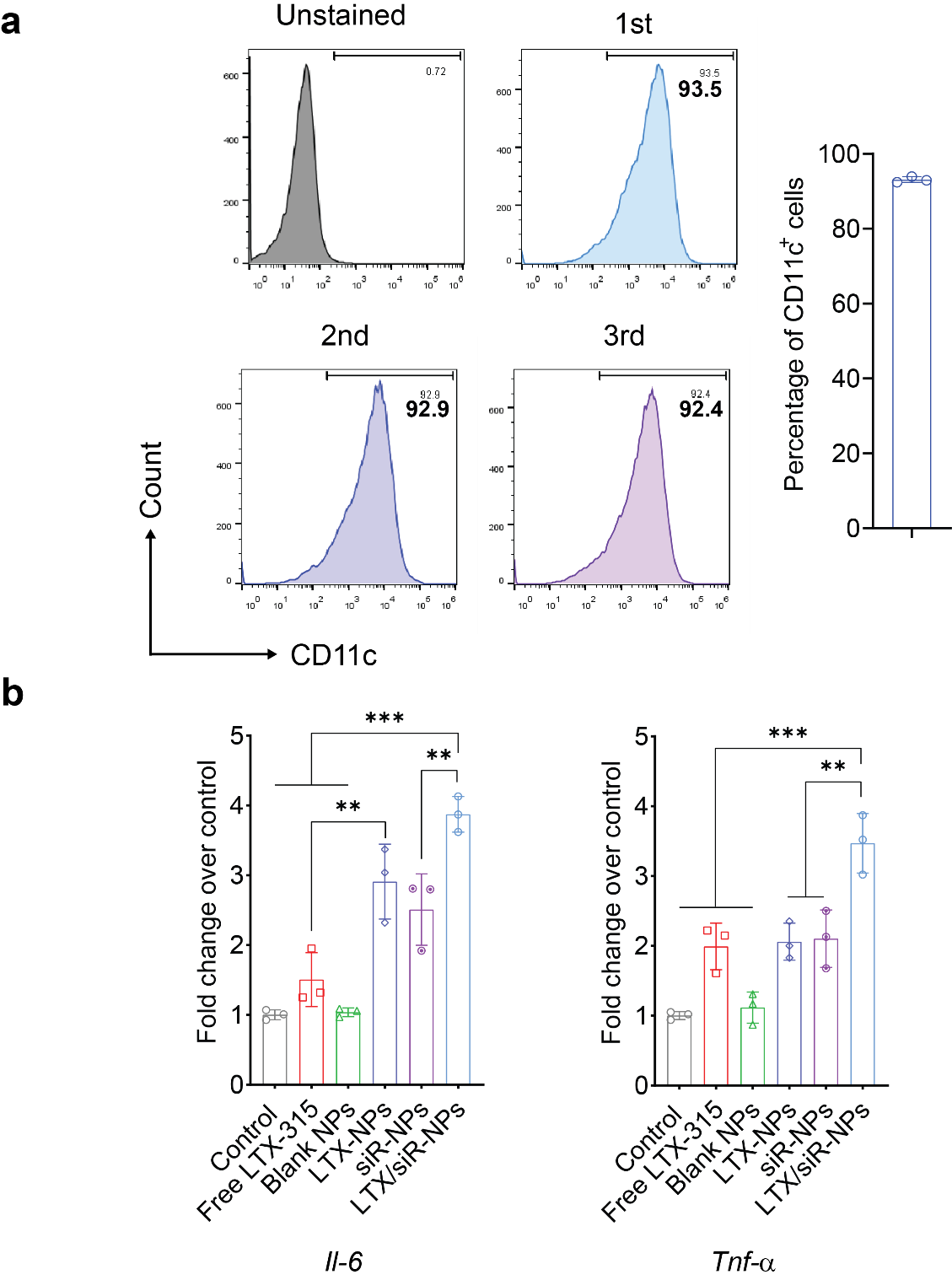


**Figure S5. (a)** The percentages of CD11c^+^ BMDCs after differentiation from mouse bone marrow cells. **(b)** The expressions of pro-inflammatory cytokines in treated BMDCs at 24h after incubation with the cultured media of treated 4T1 cells. Data were presented as mean ± SD (n=3). **, p<0.01; ***, p<0.001.


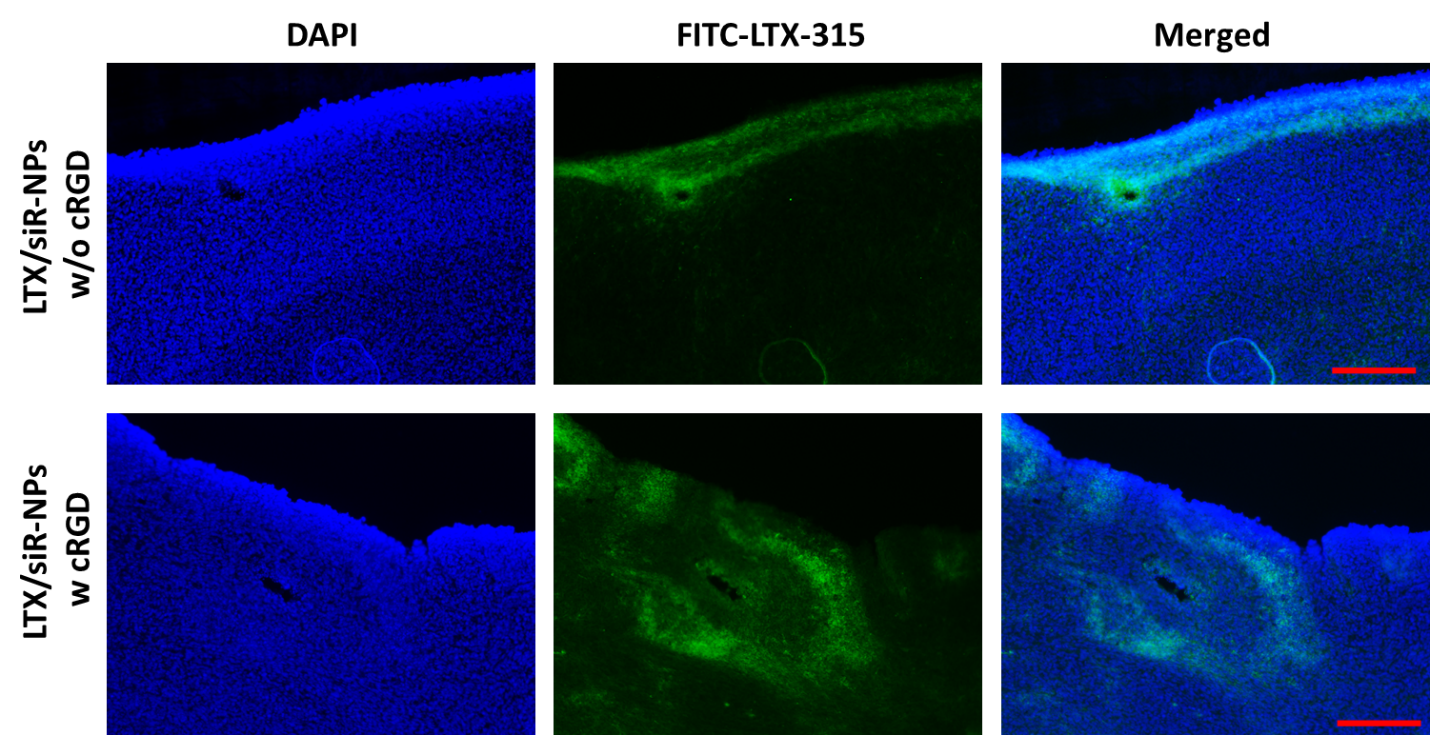


**Figure S6.** Representative images of intratumoral distribution of FITC-LTX/siR-NPs in 4T1 tumors after i.v. injection. Scale bar: 50 µm.


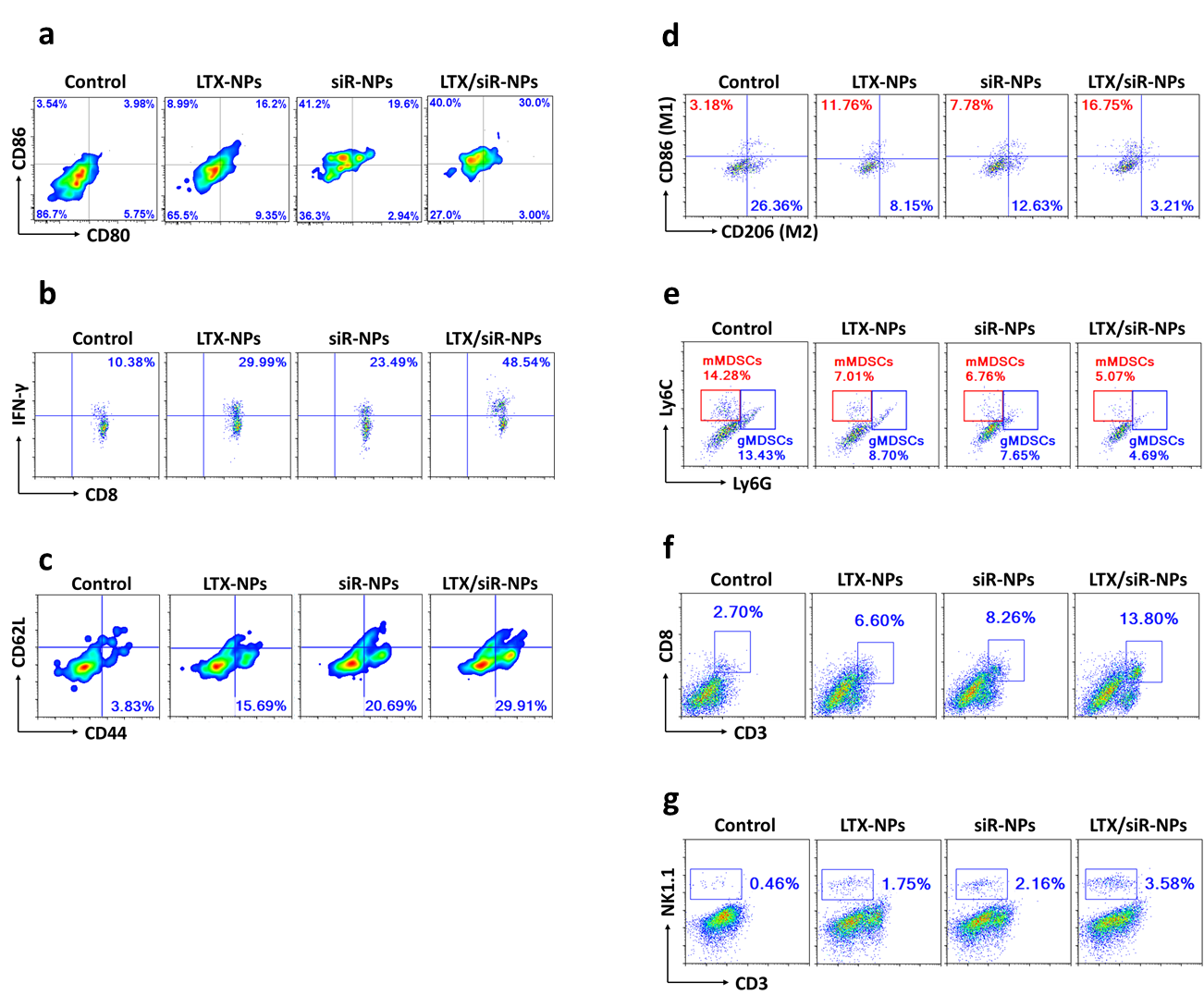


**Figure S7.** Flow cytometric analysis of (**a**) CD86^+^CD80^+^ DCs in tdLNs (gated from CD11c^+^MHCII^+^ cells), (b) CD8^+^IFN-γ^+^ T cells (gated from CD3^+^ T cells), (**c**) effector CD8^+^ T cells (CD3^+^CD8^+^CD44^+^CD62L^-^) in spleens, (**d**) intratumoral M1 (F4/80^+^CD11b^+^CD86^+^) and M2 (F4/80^+^CD11b^+^CD206^+^) macrophages, (**e**) Ly6G^+^Ly6C^-^ granulocytic MDSCs (gMDSCs) and Ly6G^-^Ly6C^+^ monocytic MDSCs (mMDSCs) (gated from CD11b^+^ cells), (**f**) cytotoxic T cells (CD3^+^CD8^+^), and (**g**) NK cells (CD3^-^NK1.1^+^).


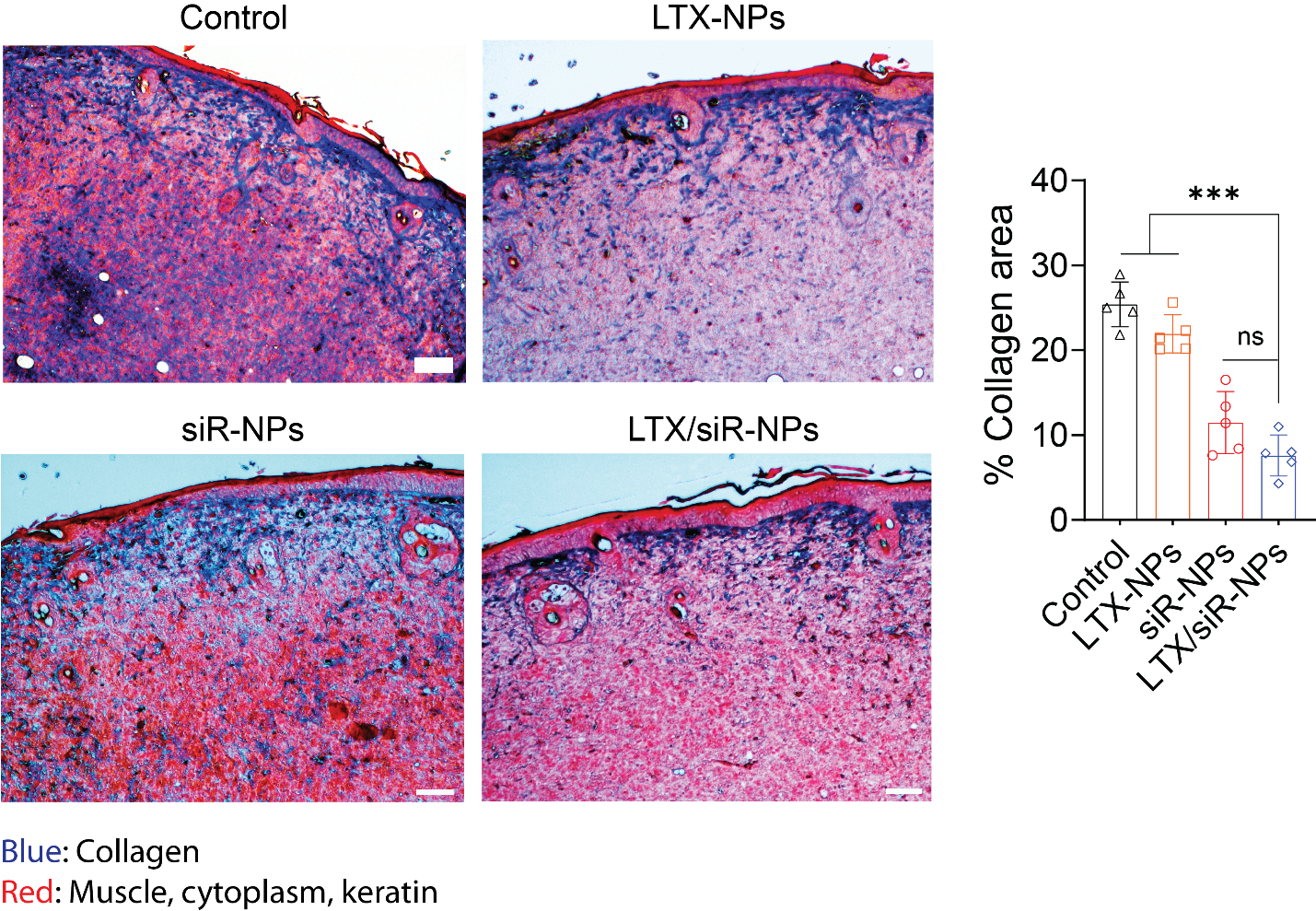


**Figure S8.** The reduced collagen densities were observed in the tumor treated with siRNA-contained NPs. Representative images and quantification of collagen distribution in 4T1 tumors determined by Masson's trichrome staining (scale bar: 20 µm). Data were represented as mean ± SD (n=5), one-way ANOVA with Tukey’s multiple comparisons test, ***, p<0.001, ns: not significant


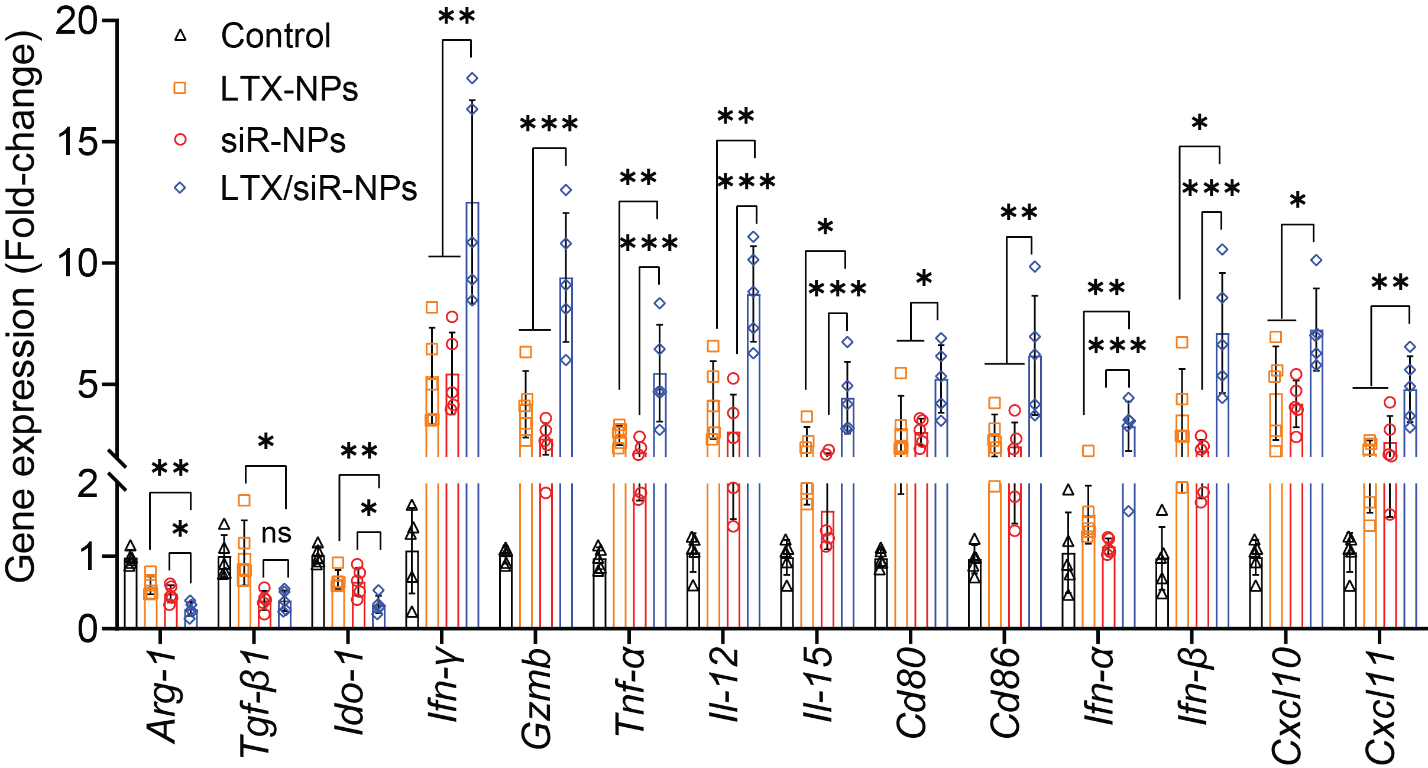


**Figure S9.** Statistical comparisons of gene expressions in the tumors treated with indicated formulations. Data represented as mean ± SD (n=5). One-way ANOVA with Tukey’s multiple comparison test, *, p<0.05; **, p<0.01; ***, p<0.001.

**
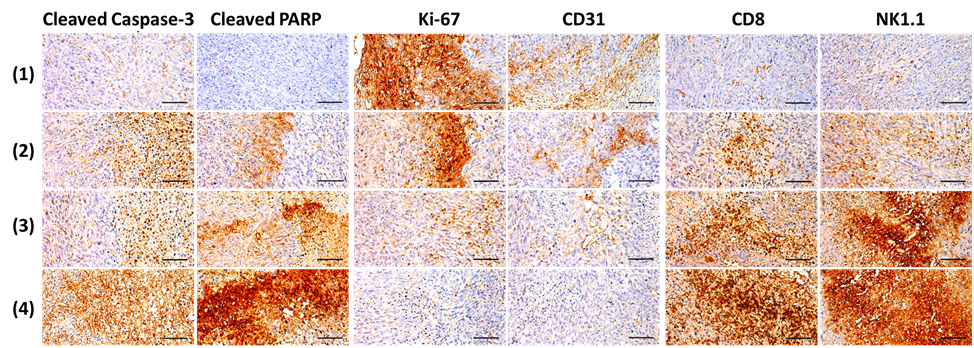
**

**Figure S10.** Immunohistochemical analysis of apoptosis markers (cleaved caspse-3; cleaved PARP), tumor proliferation (Ki-67), angiogenesis marker (CD31), and the marker of immune cells (CD8^+^ T cells and NK cells) in tumor masses following different treatments. Scale bars: 120 μm. (1) PBS (Control), (2) LTX-NPs, (3) siR-NPs, (4) LTX/siR-NPs.


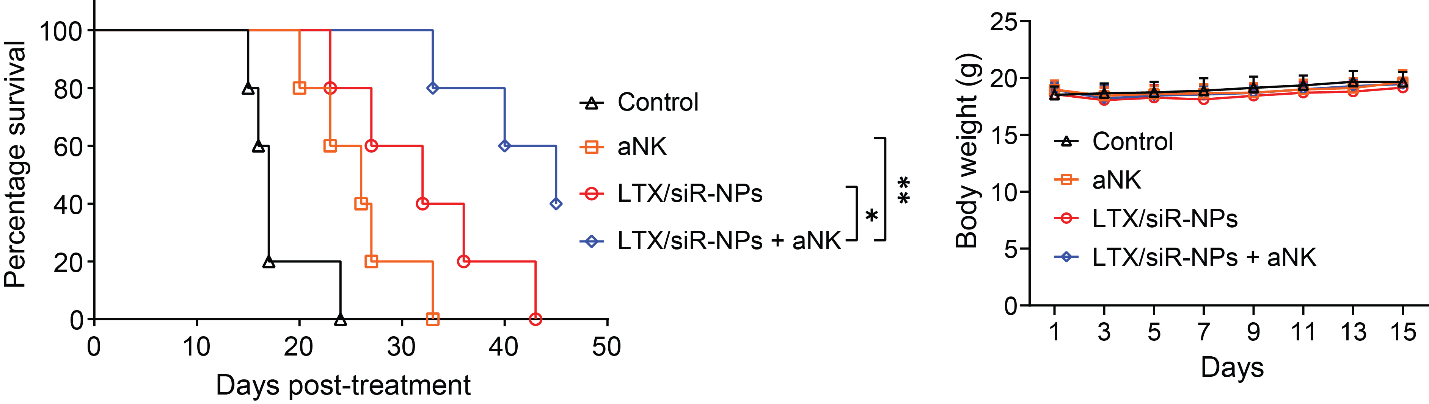


**Figure S11.** **(a)** Survival curves of 4T1 tumor-bearing mice in different treatment groups. **(b)** Body weight of treated mice.

#### Table S1. Chromatographic conditions for measuring LTX-315 concentrations

Instruments: Agilent 1100 series HPLC

Column: a reversed-phase Inertsil ODS-3 column (5 μm, 4.6 × 150 mm; GL Sciences Inc., USA)

Oven temperature: 30^o^C

Flow rate: 1 mL/min

Detector: UV at 280 nm

Injection volume: 5 μL

Diluent: acetonitrile: water = 1:1 (v/v)

Mobile phase:

- Mobile phase A: trifluoroacetic acid : water = 0.5:1000 (v/v)
- Mobile phase B: trifluoroacetic acid : acetonitrile = 0.5:1000 (v/v)

Elute mode: gradient

Elute program:

| Time (minutes) | % mobile phase A | % mobile phase B |
| --- | --- | --- |
| 0.0 | 90 | 10 |
| 8.0 | 20 | 80 |
| 13.0 | 20 | 80 |
| 13.1 | 90 | 10 |
| 16.0 | 90 | 10 |

#### Table S2. Histomorphometrical Analysis of Tumor Masses Taken form 4T1 Tumor-bearing Balb/c Mice

| Groups  Items | Control (G1) | Treatment | | |
| --- | --- | --- | --- | --- |
|  |  | G2 | G3 | G4 |
| TCV (%) | 85.09±11.39 | 53.44±6.56^a^ | 39.57±4.66^ab^ | 23.49±6.29^abc^ |
| Immunoreactive cell percentages (%/mm^2^) | | |  |  |
| Caspase-3 | 6.28±3.09 | 32.34±10.08^d^ | 53.48±5.39^de^ | 73.88±10.81^def^ |
| PARP | 4.28±2.43 | 21.15±11.38^a^ | 48.52±7.45^ab^ | 68.68±7.95^abc^ |
| Ki-67 | 71.02±10.07 | 40.15±7.25^a^ | 24.59±5.51^ab^ | 9.39±3.94^abc^ |
| CD31 | 50.79±10.53 | 30.85±5.05^d^ | 19.73±3.75^de^ | 9.14±4.22^def^ |
| Immunoreactive cell numbers (cells/mm^2^) | | |  |  |
| CD8 | 47.00±20.74 | 178.33±23.24^d^ | 364.33±113.47^de^ | 819.00±139.19^def^ |
| NK1.1 | 16.00±10.43 | 92.33±22.89^d^ | 463.67±112.02^de^ | 1120.00±177.54^def^ |

Values are expressed as mean ± SD of five tumor mass histological fields

TCV = Tumor cell volumes; PARP = Poly(ADP-ribose) polymerase; CD31 = Platelet endothelial cell adhesion molecule 1 (PECAM-1).

^a^ p<0.01 as compared with G1 by LSD test

^b^ p<0.01 as compared with G2 by LSD test

^c^ p<0.01 as compared with G3 by LSD test

^d^ p<0.01 as compared with G1 by MW test

^e^ p<0.01 as compared with G2 by MW test

^f^ p<0.01 as compared with G3 by MW test

**Table S3.** Primer list for RT-qPCR analysis

| **Gene** | **Forward sequence (5’-3’)** | **Reverse sequence (5’-3’)** |
| --- | --- | --- |
| *Arg-1* | CATTGGCTTGCGAGACGTAGAC | GCTGAAGGTCTCTTCCATCACC |
| *Tgf-β* | CAACAACGCCATCTATGAGA | TATTCCGTCTCCTTGGTTC |
| *Ido-1* | ACACGAGGCTGGCAAAGAA | TGACAAACTCACGGACTGGG |
| *Ifn-γ* | CGGCACAGTCATTGAAAGCCTA | GTTGCTGATGGCCTGATTGTC |
| *Gzmb* | TGTTTTCTCTGCCATCTGCTCTC | GCTTTGTAAAAGTCTCCAGCCTGTG |
| *Tnf-α* | CCCTCACACTCAGATCATCTTCT | GCTACGACGTGGGCTACAG |
| *Il-12* | GATGACATGGTGAAGACGGC | AGGCACAGGGTCATCATCAA |
| *Il-6* | CCTCTGGTCTTCTGGAGTACC | ACTCCTTCTGTGACTCCAGC |
| *Il-15* | GTGACTTTCATCCCAGTTGC | TTCCTTGCAGCCAGATTCTG |
| *Cd80* | CCTCAAGTTTCCATGTCCAAGGC | GAGGAGAGTTGTAACGGCAAGG |
| *Cd86* | ACGTATTGGAAGGAGATTACAGCT | TCTGTCAGCGTTACTATCCCGC |
| *Ifn-α* | GAGAAGAAACACAGCCCCTG | TCAGTCTTCCCAGCACATTG |
| *Ifn-β* | AACCTCACCTACAGGGCGGACTTCA | TCCCACGTCAATCTTTCCTCTTGCTTT |
| *Cxcl10* | GACGGTCCGCTGCAACTG | CTTCCCTATGGCCCTCATTC T |
| *Cxcl11* | AACAGGAAGGTCACAGCCATAGC | TTTGTCGCAGCCGTTACTCG |
| *Gapdh* | CTCCCACTCTTCCACCTTCG | CCACCACCCTGTTGCTGTAG |

**Table S4.** The antibody list for flow cytometry analysis

| **Anti-mouse antibody** | **Clone** | **Fluorophore** | **Company** |
| --- | --- | --- | --- |
| CD11c | N418 | APC | Biolegend |
| MHC-II | M5/114.15.2 | PerCP/Cy5.5 | Biolegend |
| CD86 | GL-1 | APC/Cy7 | Biolegend |
| CD80 | 16-10A1 | FITC | Biolegend |
| F4/80 | BM8 | PE | Biolegend |
| CD11b | M1/70 | APC | Biolegend |
| CD206 | C068C2 | PE/Cy7 | Biolegend |
| Ly6C | HK1.4 | PerCP/Cy5.5 | Biolegend |
| Ly6G | 1A8 | PE | Biolegend |
| CD3 | 17A2 | APC | Biolegend |
| CD8 | 53-6.7 | PerCP/Cy5.5 | Biolegend |
| CD44 | IM7 | PE | Biolegend |
| IFN-γ | XMG1.2 | PE/Cy7 | Biolegend |
| CD62L | MEL-14 | APC/Cy7 | Biolegend |
| NK1.1 | PK136 | PE/Cy7 | Biolegend |
| NKG2D | A10 | PE | Biolegend |
